# Focal Adhesion Kinase Provides a Collateral Vulnerability That Can Be Leveraged to Improve mTORC1 Inhibitor Efficacy

**DOI:** 10.3390/cancers14143374

**Published:** 2022-07-11

**Authors:** Leslie Cuellar-Vite, Kristen L. Weber-Bonk, Fadi W. Abdul-Karim, Christine N. Booth, Ruth A. Keri

**Affiliations:** 1 Department of Pharmacology, School of Medicine, Case Western Reserve University, Cleveland, OH 44106, USA; cuellal@ccf.org; 2 Case Comprehensive Cancer Center, School of Medicine, Case Western Reserve University, Cleveland, OH 44106, USA; 3 Department of Cancer Biology, Lerner Research Institute, Cleveland Clinic, Cleveland, OH 44195, USA; weberbk@ccf.org; 4 Anatomic Pathology, Pathology & Laboratory Medicine Institute, Cleveland Clinic, Cleveland, OH 44195, USA; fwkarim@outlook.com (F.W.A.-K.); boothc1@ccf.org (C.N.B.); 5 Department of General Medical Sciences-Oncology, School of Medicine, Case Western Reserve University, Cleveland, OH 44106, USA

**Keywords:** triple negative breast cancer, TNBC, mTORC1, FAK, focal adhesion kinase, rapamycin, defactinib, intrinsic resistance, collateral sensitivity

## Abstract

**Simple Summary:**

While the PI3K/AKT/mTORC1 pathway is highly active in breast cancer, mTORC1-targeting drugs are not effective in all breast cancer subtypes. To identify potential resistance mechanisms, we utilized a mouse model of breast cancer that continues to grow in the presence of the mTORC1 inhibitor, rapamycin. This treatment caused changes in the activity of genes that control the environment surrounding the tumor cells (the extracellular matrix or ECM). To determine if the ECM can modulate mTORC1 inhibitor effectiveness, we targeted focal adhesion kinase (FAK), an integral protein that mediates signaling from the ECM into the cell. In models that are relatively resistant to mTORC1 inhibitors, blocking FAK improved the ability of mTORC1 inhibitors to suppress tumor growth. However, in models that are sensitive to mTORC1 inhibitors, FAK suppression had no effect. These results provide preclinical evidence that the dual targeting of FAK and mTORC1 may improve therapeutic impact in cancers that are resistant to mTORC1 inhibitors.

**Abstract:**

The PI3K/AKT/mTORC1 pathway is a major therapeutic target for many cancers, particularly breast cancer. Everolimus is an mTORC1 inhibitor used in metastatic estrogen receptor-positive (ER+) and epidermal growth factor receptor 2-negative (HER2-) breast cancer. However, mTORC1 inhibitors have limited efficacy in other breast cancer subtypes. We sought to discover collateral sensitivities to mTORC1 inhibition that could be exploited to improve therapeutic response. Using a mouse model of breast cancer that is intrinsically resistant to mTORC1 inhibition, we found that rapamycin alters the expression of numerous extracellular matrix genes, suggesting a potential role for integrins/FAK in controlling mTORC1-inhibitor efficacy. FAK activation was also inversely correlated with rapamycin response in breast cancer cell lines. Supporting its potential utility in patients, FAK activation was observed in >50% of human breast cancers. While blocking FAK in mouse models of breast cancer that are highly responsive to rapamycin had no impact on tumor growth, FAK inhibition sensitized rapamycin-resistant tumors to mTORC1 inhibition. These data reveal an innate dependency on FAK when mTORC1 signaling is lost in tumors that are resistant to mTORC1 inhibitors. They also suggest a precision medicine approach to improving mTORC1 inhibitor efficacy in resistant cancers by suppressing FAK signaling.

## 1. Introduction

The PAM (PI3K/AKT/mTOR) pathway controls hallmarks of cancer such as growth, survival, and motility [1]. It is highly dysregulated across cancer types due to the accumulation of multiple genetic alterations that sustain its activation. In breast cancer, ~60% of tumors harbor genetic alterations in the PAM pathway [2]. These include activating mutations of PI3K, loss of phosphatase and tensin homolog (PTEN) or inositol polyphosphate 4-phosphatase type II (INPP4B) protein expression, and gene amplification of receptor tyrosine kinases (RTK) such as HER2 [2,3,4]. While many PAM pathway inhibitors have been evaluated, only inhibitors for three protein targets are FDA-approved in breast cancer. HER2 inhibitors, such as trastuzumab and lapatinib, are standard of care for HER2+ breast cancers [5]. Inhibitors alpelisib and everolimus, which respectively target PIK3Cα and mTORC1, are second-line therapies for advanced hormone receptor-positive/HER2-negative breast cancers [4,6,7,8]. When these inhibitors are combined with chemotherapeutic agents or hormone therapies, they increase progression-free survival in patient cohorts. However, resistance is common and leads to poor patient outcomes.

Targeting components of the PAM pathway is also often met with dose-limiting toxicities and modest efficacy [4]. Limited effectiveness is due to the multi-factorial nature and complexity of the PAM pathway, which is a network composed of numerous inputs and regulatory feedback loops [9]. Selective inhibitors of distinct members of the pathway disrupt intricate negative feedback loops. This, in turn, results in the rebound activation of other proteins that then continue to stimulate pathway signaling [10,11]. In some cases, breast cancer cells may also have intrinsically higher activation or expression of pathway proteins that prevents even an initial response to therapy. While elucidating the many components and modulators of the PAM pathway has garnered much attention, pathways that provide alternative mechanisms for sustaining growth in the absence of PAM signaling have not been sufficiently explored. Identifying such pathways should provide new protein targets for additional monotherapies or drug combinations that enhance the efficacy of PAM-pathway inhibitors as well as usurp resistance pathways.

Mechanisms of resistance to PAM pathway inhibitors has been characterized as intrinsic, adaptive, or acquired, with most maintaining the activation of the pathway [12]. For example, activating mutations in the *PI3KCA* gene and the activation of the cyclin D1:CDK4/6 signaling pathway can each sustain PAM pathway signaling, even in the presence of HER2 or mTORC1 inhibitors (mTORC1i) [4,5,6]. While targeting these additional activating mechanisms can provide clinical impact, response is typically transient due to the many routes available to stimulate this core pathway. An alternative approach to identifying mechanisms that sustain PAM signaling is to discover growth-promoting signals that operate independently of the PAM pathway but provide collateral sensitivity. As a concept that initially emerged from antibiotic resistance, collateral sensitivity occurs when a cell is treated with inhibitor A and, as a result, becomes hypersensitive to inhibitor B, with A and B targeting different proteins/pathways [13]. In cancer, targeting ABC transporters is a collateral sensitivity to multidrug-resistant cancers previously treated with paclitaxel, doxorubicin, or other inhibitors [13,14]. Other studies have demonstrated collateral sensitivity to taxanes in the context of resistance to PRMT5 inhibitors or dependency on MEK/EGFR in chemoresistant tumors [15,16]. Discovering collateral pathways that continue to drive tumor growth in the presence of PAM inhibitors should reveal new targets whose blockade could potentiate PAM inhibitor efficacy in resistant tumors. An area that has not been fully explored is the role of microenvironmental factors, such as the extracellular matrix (ECM), in controlling the efficacy of PAM inhibitors.

In cancer, the ECM facilitates growth, migration, and invasion [17]. It is comprised of growth factors, as well as enzymatic, structural, and matricellular proteins [18]. Signal transduction from the ECM to the cell is primarily mediated by integrins. When bound to ECM components, various alpha and beta integrin subunits heterodimerize at the plasma membrane and recruit multiple proteins to form focal adhesions [17,19]. Focal adhesion kinase (FAK) is a primary intracellular mediator that localizes to integrins and is autophosphorylated. Phosphorylated residues in FAK (autophosphorylation sites and sites that are phosphorylated by Src family kinases or SFKs) serve as docking domains for other proteins involved in various downstream cell signaling pathways [20]. FAK can mediate resistance to upstream inhibitors of the PAM pathway in both liquid and solid tumors. Through a systematic analysis, Schwill and others identified FAK as a major signaling node that is activated as an adaptive response to various HER2 inhibitors in breast cancer [21]. In addition, the HER2 inhibitor lapatinib increases FAK autophosphorylation [22]. Trastuzumab- and lapatinib-resistant breast cancer cells also increase FAK phosphorylation [23,24]. In cancers other than breast, PAM inhibitors can increase integrin expression, FAK autophosphorylation, or both [25,26,27]. Studies in pancreatic neuroendocrine tumors and acute lymphoblastic leukemia have further found that the dual inhibition of mTORC1 and FAK, through pharmacological or genetic silencing approaches, decreases cell growth [28,29,30]. As indicated above, mTORC1i (e.g., everolimus) are approved as a second line of therapy in luminal breast cancer. mTORC1i are less efficacious in other breast cancer subtypes, such as triple negative breast cancer (TNBC) [31,32]. It is unknown if cells that are resistant to mTORC1i, like TNBC, rely on FAK for sustained growth. If so, this would suggest that FAK inhibitors may be able to reinstate mTORC1 sensitivity and provide a therapeutic avenue for mTORC1i in TNBC and other resistant cancers.

Herein, we describe an approach for discovering alternative pathways that sustain growth in the presence of mTORC1i. We found that mTORC1 inhibition with the prototypical inhibitor, rapamycin, in a TNBC xenograft model is able to suppress mTORC1 signaling but does not fully block tumor growth. Moreover, rapamycin alters the expression of a large cohort of extracellular matrix genes, suggestive of ECM remodeling and the potential dependency of these tumors on integrin signaling. Since FAK is a common downstream signaling node of ECM proteins that is therapeutically targetable [33], we asked whether FAK inhibitors may have utility in improving mTORC1i response. We found that FAK is active across all clinically defined breast cancer subtypes and that its activity is correlated with patient outcomes in the group as a whole, as well as in TNBC. Importantly, resistance to mTORC1i is associated with increased FAK activity in cell line models of breast cancer, and rapamycin treatment in vivo increased the expression of genes comprising a signature of the FAK-inhibitor response. In two preclinical mouse models of breast cancer wherein rapamycin is only modestly effective, FAK inhibition alone did not affect tumor growth. However, combining a FAK inhibitor with rapamycin decreased tumor growth to a greater extent than either drug alone. In contrast, in two tumor models that are highly responsive to mTORC1i, FAK blockade had no effect on rapamycin efficacy. Together, these results illustrate that, upon mTORC1 inhibition within an intact tumor microenvironment, FAK signaling can sustain breast cancer growth in tumors and convey resistance to mTORC1i. Thus, FAK provides a collateral sensitivity in tumors that are resistant to mTORC1 suppression. These data provide foundational support for the dual use of mTORC1 and FAK inhibitors in breast cancers that are resistant to rapamycin analogs or rapalogs.

## 2. Methods

### 2.1. Cell Culture

SUM149 and SUM159 were purchased from Asterand Bioscience (Detroit, MI, USA). All other cell lines were purchased from the American Type Culture Collection (ATCC, Manassas, VA, USA). BT474 cells were grown in Hybri-Care Media (ATCC); BT-549, HCC39, HCC70, MDA-MB-231, MDA-MB-453, MDA-MB-468, T47D, ZR-75-10 in RPMI 1640 (Corning, Corning, NY, USA); MCF7 and MDA-MB-453 in DMEM (Corning); and SK-BR-3 in McCoy’s 5A (Sigma-Aldrich, St. Louis, MI, USA). Media was supplemented with 10% FBS (Atlanta Biologicals, Flowery Branch, GA, USA) and 1% penicillin–streptamycin (Gibco, Waltham, MA, USA). SUM149 and SUM159 cells were grown in Ham’s/F12 (Gibco), supplemented with 5% FBS, 1% penicillin–streptamycin, 1M HEPES (Gibco), and 1 mg/mL hydrocortisone (Sigma-Aldrich). All cell lines were maintained at 37 °C with 5% CO_2_. All cell lines were confirmed to be free of mycoplasma contamination (Bimake, Houston, TX, USA or Lonza, Basal, Switzerland) every 2 months.

### 2.2. Drugs

Rapamycin (LC Laboratories, Woburn, MA, USA; R-5000) and defactinib/VS-6063 (ChemieTek, Indianapolis, IN, USA; 1073154-85-4) were dissolved in DMSO (Fischer Chemical, Zurich, Switzerland) for all in vitro experiments. For in vivo studies, rapamycin was dissolved in 5.2% Tween80/5.2% polyethylene glycol in 0.9% saline and defactinib in 50% propylene glycol/50% sterile water.

### 2.3. In Vivo Studies

All in vivo experiments were approved by the Institutional Animal Care and Use Committee at Case Western Reserve University or at the Cleveland Clinic Lerner Research Institute. Mice were housed in microisolator units maintained on a 12 h light/dark cycle. Mice were given standard chow and water. Tumor pieces from MMTV-NeuT, MMTV-PyMT, and TM00089, or MDA-MB-231 cells were orthotopically xenografted in both inguinal mammary fat pads of adult female FVB/N or NOD/scid/γ (NSG) mice, depending on the tumor species of origin. MMTV-NeuT and MMTV-PyMT tumor banks were generated as previously published [34]. Tumor response was independent of being in the same host mouse (data not shown). Patient-derived xenograft TM00089 was purchased from The Jackson Laboratory, Bar Harbor, ME, USA. For the 3 day short-term treatment, mice with palpable tumors (200–300 mm^3^) were randomized into vehicle- and rapamycin-treated groups. Rapamycin was injected intraperitoneally at 7.5 mg/kg on days 1 and 3. 1.5 h after the second injection, tumors were harvested. Each tumor was cut in half; one half was placed in RNA later (Thermo Fisher Scientific, Waltham, MA, USA) and the second half in RIPA lysis buffer (1% NP-40, 0.25% sodium deoxycholate, 0.10% SDS, 0.15 M NaCl, 0.05 M Tris-HCL pH 7.4, 0.002 M EDTA) supplemented with sodium orthovanadate, protease inhibitors, and PhosSTOP (Roche, Basel, Switzerland).

For long-term treatments, mice with palpable tumors (~300 mm^3^) were randomized into four treatment groups: vehicle, rapamycin alone, defactinib alone, or the combination. Rapamycin was injected, intraperitoneally (ip), every other day at 7.5 mg/kg. Defactinib was suspended in 50% propylene glycol/50% sterile water and administered by oral gavage daily at 50 mg/kg. Tumor size was measured by calipers twice weekly and volume was calculated as (length × width^2^)/2. To assess overt toxicity, mouse weight was measured once per week.

### 2.4. Protein Collection and Western Blots

Tumors in RIPA lysis buffer (with the above listed supplements) were homogenized using a rotor stator homogenizer for 30 s to 1 min. Samples were stored overnight at −80 °C and then spun at 10,000 rpm for 10 min at 4 °C the next day to remove debris. For collecting protein from cell lines, the cells were washed twice with ice-cold PBS and then incubated in lysis buffer with supplements. Cells were then scraped and transferred to Eppendorf tubes and incubated for 30 min on ice. Tubes were spun at 10,000 rpm for 10 min at 4 °C. The protein-containing supernatant was quantified (Bradford Assay, Bio-Rad, Hercules, CA, USA), diluted in reducing buffer (2× Laemmli Sample Buffer, Bio-Rad), and boiled. Protein lysates (60 μg) were loaded onto precast polyacrylamide gels (Novex 4–20% Tris-Glycine Mini Gels, Thermo Fisher Scientific). Proteins were transferred from the gels onto low-fluorescence PVDF membrane (MilliporeSigma, Burlington, MA, USA), stained for 5 min with REVERT total protein stain (LI-COR Biosciences, Lincoln, NE, USA), washed with REVERT wash solution, and imaged on an Odyssey or FC Imagers (LI-COR Biosciences) for total protein. Membranes were then washed in TBST (10 mM Tris-HCl pH7.5, 154 mM NaCl, 0.05% Tween 20), and blocked for 1 h with 5% non-fat dried milk and 5% bovine serum albumin (BSA) in TBST. After TBST washes, membranes were cut through the 90 kDa and 50 kDa bands of the Chameleon Duo Pre-stained Protein Ladder (LI-COR Biosciences). These cuts insured that at least one ladder band was above and below the protein of interest. Membranes were incubated overnight at 4 °C on a rocker with primary antibodies diluted in 5% BSA in TBST as follows: phosphorylated Y397-FAK (Cell Signaling Technology, Danvers, MA, USA; 8556) 1:150, total FAK (Invitrogen, Waltham, MA, USA; AHO1112) 1:1500, phosphorylated S235/236–S6 (Cell Signaling Technology, 4858) 1:2000, total S6 (Cell Signaling Technology, 2317) 1:100, β-Actin (Sigma-Aldrich, A2228) 1:10,000, and vinculin (Sigma-Aldrich, V9131) 1:1000.

The next day, membranes were washed three times for 5 min in TBST and probed with secondary antibodies, either anti-rabbit 800CW (LI-COR Biosciences) or anti-mouse 680RD (LI-COR Biosciences), at 1:10,000 dilutions in 5% fat dried milk in TBST for 1 h, rocking in the dark at room temperature (RT^o^). After TBST washes, membranes were imaged. All membranes were first probed and imaged for phosphorylated proteins, washed in TBST three times for 20 min, and then probed for the respective parent/total protein member. Densitometry was performed using Image Studio (LI-COR Biosciences).

### 2.5. RNA Isolation and RNA-seq

Total RNA was isolated from MDA-MB-231 xenograft tumors per the manufacturer’s protocol (RNeasy Plus Mini Kit, Qiagen, Hilden, Germany). Quality control, library construction, and sequencing were performed by Novogene Corporation Inc. The Illumina platform was utilized to generate paired-end reads. Raw files were processed and analyzed by Partek Flow. Reads were aligned to the Hg38 genome with STAR, and differential expression was performed by DESeq2. Genes were deemed differentially expressed with a false discovery rate (FDR) set to <0.5. PCA analysis was completed using Partek Flow software.

### 2.6. Real-Time Quantitative RT-PCR

Complementary DNA was generated using SuperScript IV (Invitrogen, Thermo Fischer Scientific). Quantitative real-time PCR was performed using a QuantStudio3 (Applied Biosystems, Waltham, MA, USA). Taqman gene expression assays (Applied Biosystems) for human *FN1* (Hs00365052_m1), *COL8A2* (Hs078287101_m1), *COL7A1* (Hs00164310_m1), *TNC* (Hs01115665_m1), and *GAPDH* (Hs02758991_g1) were utilized.

### 2.7. Patient Tumor Samples

Paraffin-embedded breast cancer sections from sixty de-identified patients with corresponding receptor-defined subtype information (ER+/PR+/HER2-, ER+/PR+/HER2+, ER-/PR-/HER2+, and ER-/PR-/HER2-) were obtained from the University Hospitals Cleveland Medical Center Department of Pathology archives. Tissue microarrays (TMA) of 157 paraffin-embedded breast cancers and cell lines of known subtype were generated using archival samples obtained from University Hospitals Cleveland Medical Center [35]. All tumors on the TMA were linked to an Institutional Review Board (IRB)-approved database including clinicopathological information and clinical follow-up data.

### 2.8. Immunohistochemistry and Scoring

All sections were deparaffinized and rehydrated. For antigen retrieval, slides were incubated in a decloaking chamber (Biocare Medical, Pacheco, CA, USA) for 10 min at 125 °C in citrate buffer (1.8 mM citric acid, 8.2 sodium citrate, pH 6.0). Slides were then washed in TBST and blocked with 5% BSA in TBST for 1 h. After two TBST washes, slides were incubated in primary antibody in 5% BSA and 15 μL/mL normal goat serum in TBST overnight at 4 °C. Patient samples (whole sections and TMAs) were incubated with anti-phosphorylated Y397-FAK antibody (Invitrogen, 700255) at a 1:1000 dilution. The next day, slides were washed in TBST and incubated with secondary antibody for 1 h at RT^o^, utilizing the Dako Envision Plus Rabbit HRP Kit (Dako, Glostrup, Denmark). Slides were incubated in 3,3′-diaminobenzidine (DAB) for 1 min to detect the bound antibody. Slides were then counterstained with Gill’s hematoxylin 3 (Thermo Fisher Scientific), followed by Scott’s Bluing Solution. Lastly, slides were dehydrated and mounted with Permount (Thermo Fisher Scientific).

H-scores were generated by 2 independent board-certified pathologists (FWAK and CNB) [34]. Scores were based on the percentage of tumor cells that were positive for staining (10% = 1, 20% = 2, 30% = 3, etc.) and staining intensity (1+, 2+, 3+). The final score was calculated as: (percent positive staining) × (staining intensity), with the highest value being 30. The association of pFAK staining with clinical outcomes was assessed by calculating Kaplan–Meier survival probabilities that were compared with the log-rank test. The pFAK positive group included all patients whose tumors had an H-score ≥ 1. Images were collected at 10× magnification using a Nikon Opti-Phot-2 microscope, Axiocam, and Axiovision software (Zeiss, Jena, Germany).

### 2.9. Cell Growth Assays

Breast cancer cell lines were seeded and treated the following day with vehicle or rapamycin (1 μM) in some experiments, or with vehicle, rapamycin (250 nM–1 μM), defactinib (250 nM–1 μM), or the combination (250 nM–1 μM, rapamycin and defactinib at 1:1 ratios). Relative cell number was assessed 3 days after initial treatment. Cells were washed with PBS and fixed/stained with crystal violet (0.05% crystal violet, 1% formaldehyde, and 1% methanol in PBS) for 20 min at RT^o^. Wells were washed with tap water, and plates were dried overnight at room temperature. To quantify staining, 2.5% acetic acid in sterile water was added to each well. Optical density at 590 nm absorbance was determined using a VERSAmax (Molecular Devices, San Jose, CA, USA) plate reader.

### 2.10. Bioinformatics

All pathway analyses were performed in ConsensusPathDb using over-representation analysis [36,37]. Gene-set enrichment analysis (GSEA) was used to assess enrichment of the extracellular matrix organization and the FAK-regulated gene signature from the short-term rapamycin-treated MDA-MB-231 tumors [38,39].

Spearman’s correlation coefficients of total S6 protein, S6 pS235/236, total 4E-BP1, 4E-BP1 S65, total p70S6K, and p70S6K T389 (RPPA) to mRNA expression (RNA-seq) in 876 patients was generated using The Cancer Genome Atlas (TCGA) Pan-Cancer Atlas dataset [3,40], accessed through CBioPortal [41,42]. Genes were sorted from highest Spearman’s correlation coefficient to lowest. The top and bottom 10% of these genes were then further analyzed for pathway enrichment.

To create the FAK-dependent gene signature (FDS), dataset GSE61756 was analyzed with NCBI’s GEO2R web tool. SK-BR-3 transcriptomes from cells that were untreated or treated for 24 h with FAK inhibitors, Y15 (1 μM), or PF-04554878/defactinib (1 μM), were analyzed. Differentially expressed genes from both treatment groups were identified with an adjusted *p*-value < 0.05 and log fold change < 0.5. Overlapped genes from the two treatment groups (58 genes) were used to generate a FAK-dependent gene signature. Summation of the 58 genes in the FDS were calculated for each patient from The Cancer Genome Atlas (TCGA) Pan-Cancer Atlas dataset. To assess the association of the FDS with patient outcomes, the top 5% of patients (*n* = 54) with high FDS were compared to the bottom 95% of patients (*n* = 1028). Kaplan–Meier curves of overall survival were generated by GraphPad and compared with the log-rank test.

### 2.11. Statistical Methods

Statistical analyses were performed using two-tailed Student’s *t*-tests (in vitro assays), a Mann–Whitney U test (mouse tumor growth response), or a log-rank test (Kaplan–Meier curves). All in vitro experiments were performed at least three times, each with 3 technical replicates. The mean of the technical replicates is shown, and variability is indicated by standard deviation. *p*-values < 0.05 are considered statistically significant.

## 3. Results

### 3.1. Inhibition of mTORC1 Signaling in Resistant Tumors Modulates the Expression of Extracellular Matrix Genes

**Figure 1:**
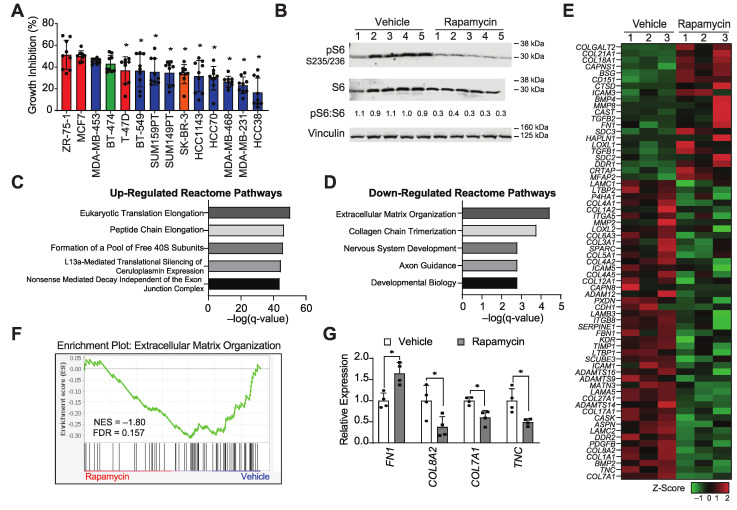
Inhibition of mTORC1 signaling in resistant tumors reveals an association with the extracellular matrix. (**A**) Relative growth inhibition of breast cancer cell lines in response to 1 μM rapamycin after 3 days of treatment. Each cell line was compared to their vehicle (DMSO) control (set to 1, not shown). Data is expressed as average ± STDEV (* = *p* < 0.05 compared to ZR-75-1 cells); *n* = 3 independent experiments in triplicate. Different colors represent different receptor-defined subtypes: red, ER+/PR+/HER2-, green, ER+/PR+/HER2+, orange, ER-/PR-/HER2+, blue, ER-/PR-/HER2-. (**B**) Representative western blots examining levels of phosphorylated S6 (pS6 S235/236), total S6 (S6), and vinculin (loading control); the quantitation of the pS6 to S6 ratio is shown. Total protein loading is shown in Supplementary Figure S1A. Numbers indicate different tumors. Reactome pathway enrichment analysis of the (**C**) differentially up- and (**D**) down-regulated genes in MDA-MB-231 xenografted tumors following 3 days of rapamycin treatment. (**E**) Heatmap and (**F**) GSEA plot of differentially expressed genes (FDR < 0.5) in the reactome pathway: extracellular matrix organization. (**G**) Confirmation of gene expression changes for *FN1*, *COL8A2*, *COL7A1*, and *TNC* in MDA-MB-231 orthotopic xenografts in response to rapamycin. Data are expressed as average ± SD (* = *p* < 0.05 compared to respective vehicle); *n* = 4 independent tumors.

Breast cancer subtypes exhibit a range of sensitivities to mTORC1i [31,32]. To discover novel mechanisms of resistance, we first identified cell line models that have minimal growth inhibition in response to rapamycin, a well-established mTORC1 inhibitor. We evaluated growth response after three days using cell lines representing diverse breast cancer subtypes (Figure 1A) [43,44]. TNBC cell lines HCC38, MDA-MB-231, and MDA-MB-468 cells were the least growth-inhibited in this cohort. MDA-MB-231 cells represent the highly aggressive claudin-low subtype of breast cancer and readily form tumors when implanted orthotopically in the mammary fats of NSG mice [45,46]. We used these cells to elucidate potential therapeutically targetable, in vivo collateral sensitivities that occur in tumors that are resistant to mTORC1i. Mice harboring established orthotopic xenografts were treated with rapamycin for three days. As a pharmacodynamic marker for mTORC1 inhibition, the reduced phosphorylation of the mTORC1 downstream target, S6, was confirmed (Figure 1B; Supplementary Figure S1A,B). These data indicate that rapamycin is highly effective for blocking mTORC1 activity even though the cells are relatively resistant to growth inhibition by this drug in vitro. RNA-seq and principal components analysis of vehicle- and rapamycin-treated tumor replicates revealed consistent responses in principal component 2, with tumors clustering according to their respective treatment groups (Supplementary Figure S1C). This analysis revealed 3696 consistently differentially expressed genes in response to rapamycin in mTORC1-resistant tumors (FDR cut-off < 0.5, Supplementary Table S1), again indicating that rapamycin has effects on tumor cells independent of its ability to suppress in vitro growth. Genes induced by rapamycin were associated with translation, possibly as a compensatory mechanism in response to the known ability of rapamycin to block this function (Figure 1C). In contrast, the top reactome pathway for down-regulated genes was extracellular matrix organization (Figure 1D). Indeed, of the differentially expressed genes, more than half of the genes in the ECM signature were down-regulated upon rapamycin treatment (Figure 1E). This is further illustrated by gene-set enrichment analysis (GSEA), wherein the pathway is enriched in the vehicle-treated tumors (Figure 1F), i.e., they are repressed in response to rapamycin. We further confirmed rapamycin-induced changes in the expression of a subset of these genes by real-time quantitative PCR (Figure 1G). These data indicate that the inhibition of mTORC1 with rapamycin impacts the extracellular matrix and may implicate the ECM as a mechanism that modulates response to mTORC1i.

### 3.2. The Extracellular Matrix and FAK Activation Are Associated with Resistance to mTORC1 Inhibitors

**Figure 2:**
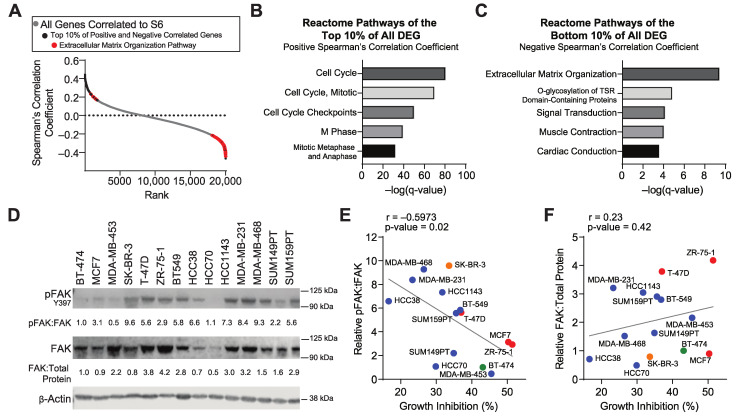
The extracellular matrix and FAK activation are associated with resistance to mTORC1 inhibitors. (**A**) Ranking of genes by their Spearman’s correlation coefficient to S6 (RPPA) from 876 breast cancer patients in the TCGA, Pan-Cancer Atlas dataset. Red dots are genes that comprise the ECM remodeling Reactome pathway. Reactome pathway enrichment analysis of the (**B**) top and (**C**) bottom 10% of genes correlated with S6 protein in breast cancer. (**D**) Protein expression of phosphorylated FAK (pFAK Y397), total FAK (FAK), and β-actin (loading control) in 14 breast cancer cell lines. Quantitation of pFAK to FAK and FAK to total protein (Supplementary Figure S2) are shown. (**E**) Scatterplot illustrating the correlation between the pFAK:FAK ratio to rapamycin growth inhibition. r = Pearson’s correlation coefficient, two-tailed *p*-value. Colors represent breast cancer receptor-defined subtypes: red, ER+/PR+/HER2-, green, ER+/PR+/HER2+, orange, ER-/PR-/HER2+, blue, ER-/PR-/HER2-. (**F**) Scatterplot illustrating the correlation between relative rapamycin growth inhibition and the calculated FAK/total protein ratio.

Analysis of the MDA-MB-231 human cell line xenograft revealed a link between mTORC1 signaling and the expression of ECM-modulating genes. To determine if ECM genes are also associated with mTORC1 signaling in breast cancer patient samples, we interrogated the TCGA, Pan-Cancer Atlas dataset [3,40]. We first ranked the correlation of all expressed genes with total S6 protein using Spearman’s correlation coefficient and then assessed pathway enrichment in the top and bottom 10% of correlated genes (Figure 2A–C). The positively correlated genes (top 10%) were found to be enriched in cell cycle pathways. On the other hand, the extracellular matrix organization pathway was the top pathway for the negatively correlated genes (the bottom 10%). We additionally assessed the pathways of the top 10% of negatively and positively correlated genes with S6 pS235/236, 4E-BP1, 4E-BP1 S65, p70S6K, and p70S6K T389, additional downstream components of the mTORC1 signaling pathway (Supplementary Tables S2–S6). Extracellular matrix organization was amongst the top reactome pathways for negatively correlated genes for 4E-BP1 and p70S6K. In contrast, pathways that were negatively correlated with phosphorylated S6, 4E-BP1, and p70S6K included protein translation, insulin-like growth factor uptake, and cell cycle, but the ECM signature was not identified for the phosphorylated isoforms of these proteins. This is likely due to differences in time scales for phosphorylation versus the gene expression of ECM components. Moreover, the patients in this analysis were not treated with mTORCi. The consistent negative association of the core components of mTORC1 signaling (S6, p70S6K, and 4E-BP1) with ECM gene expression signatures supports the possibility that chronic mTORC1 inhibition may result in a reorganization of the ECM and that the tumor cells could benefit from novel interactions derived from the ECM.

Integrins are hub receptors for ECM structural proteins such as collagens and fibronectin [47]. There is substantial complexity in ECM/integrin interactions, making it difficult to discern the impact of each on tumor growth and drug response. However, focal adhesion kinase (FAK) is a major downstream mediator of integrin signaling that is therapeutically targetable [48]. To determine if blocking FAK activity may provide an opportunity to enhance responsiveness to mTORC1i, we first determined if activated FAK is associated with intrinsic mTORC1i responsiveness. We quantified basal protein levels of total FAK and phosphorylated/activated FAK (Y397) in the fourteen breast cancer cell lines for which we had already assessed growth inhibition in response to rapamycin (Figure 2D and Supplementary Figure S2). We then compared the extent of the association of FAK and pFAK to rapamycin growth inhibition in each breast cancer cell line. The levels of phosphorylated/activated FAK were significantly inversely correlated with rapamycin response (r = −0.6), with MDA-MB-231 cells having one of the highest levels of basal pFAK and the lowest growth responses to rapamycin (Figure 2E). These results illustrate that the high basal activation of FAK is correlated with a lower response to mTORC1i. To determine if this was specific to FAK activation, we also assessed the association of total FAK protein with rapamycin responsiveness. In stark contrast to pFAK, there was no statistically significant correlation of total FAK protein with rapamycin response (Figure 2F), indicating that FAK activation is highly associated with resistance to mTORC1i.

### 3.3. FAK Activation Is Associated with Poor Breast Cancer Patient Outcomes

**Figure 3:**
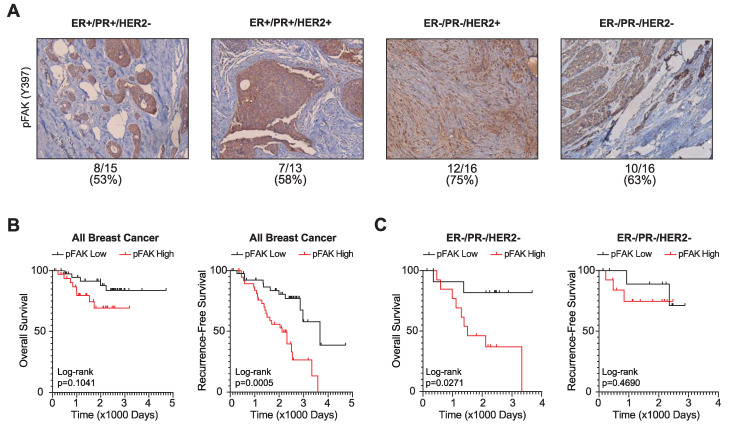
FAK activation is associated with worse breast cancer patient outcomes. (**A**) Representative images (10×) of 60 primary breast cancer tissue cohorts grouped by subtype. Tissues were stained for phosphorylated FAK on tyrosine residue 397 (pFAK Y397). ER, estrogen receptor. PR, progesterone receptor. HER2, human epidermal growth factor receptor 2. Below: Calculated percentage of positively stained tumors. (**B**) Overall and recurrence-free survival outcomes of stained tissue microarray for pFAK Y397 in all breast cancer subtypes. The top 30% of patients with high pFAK Y397 (*n* = 40) were compared to the bottom 30% of patients with low pFAK Y397 (*n* = 40). Log-rank test where *p* < 0.05 is statistically significant. (**C**) Overall and recurrence-free survival in TNBC patients comparing the top 30% with high pFAK Y397 (*n* = 13) to the bottom 30% with low pFAK Y397 (*n* = 13).

The above studies suggest that FAK may be a key driver of breast cancer growth that modulates responsiveness to mTORC1i. To evaluate the extent to which FAK is activated in human tumors, we used immunohistochemical staining for phosphorylated/activated FAK (Y397) and assessed the association of its expression with receptor subtypes or patient outcomes in two different cohorts (Figure 3). In the first cohort, sixty primary tumor samples were immunostained for pFAK. This revealed that >50% of tumors express activated FAK, independent of subtype (Figure 3A and Supplementary Figure S3). In a second cohort that included patient-outcome data, a tissue microarray containing 150 breast cancer patient samples revealed that high levels of pFAK are associated with worse recurrence-free survival and trend towards worse overall survival across the entire breast cancer cohort, including all receptor-defined subtypes (Figure 3B,C). When specifically examining the TNBC subtype, high pFAK is associated with worse overall survival and a trend towards association with worse recurrence-free survival in TNBC (ER-/PR-/HER2-), in concordance with data from Shen et al. examining the association of pFAK with disease-free and distant-metastasis-free survival with a different patient cohort [49]. Together, these data indicate that FAK is activated (phosphorylated) in many breast cancers and is associated with worse patient outcomes.

### 3.4. Pharmacologically Inhibiting mTORC1 Activity Increases the Expression of a FAK Signature Gene Set

The induction of ECM gene expression in response to rapamycin coupled with the inverse correlation of pFAK and mTORC1 activity suggested that the suppression of the mTORC1 pathway may reveal a reliance on FAK signaling for sustained growth. To determine if mTORC1 inhibition induces FAK pathway activity in tumors, we assessed the impact of rapamycin on the expression of a set of FAK-dependent genes. We developed a FAK-dependent gene signature (FDS) by interrogating a publicly available microarray data set (GSE61756) that quantified transcriptome changes in response to small-molecule inhibitors of FAK. Fifty-eight intersecting genes were identified that were significantly down-regulated (*p* < 0.05) by FAK inhibitors (i.e., FAK-dependent) in SK-BR-3 cells treated with Y15 or PF-04554878/VS-6063 (defactinib) (Supplementary Figure S4A,B). Pathway analysis revealed that the constituent genes within the FDS are associated with p53 regulation of the cell cycle, cell death, ErbB2/HER2 signaling, and RNA metabolism (Supplementary Figure S4C), indicating that FAK-dependent genes are essential for survival and proliferation.

**Figure 4:**
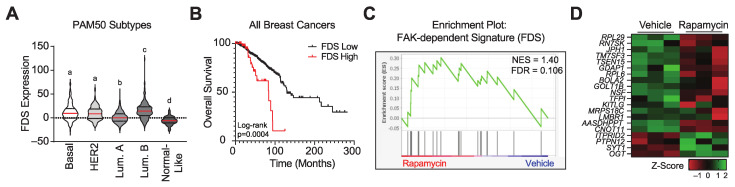
Pharmacologically inhibiting mTORC1 activity leads to the increased expression of a FAK signature gene set. (**A**) Analysis of FDS expression in breast cancer subtypes using data from the TCGA Pan-Cancer Atlas. Groups with the same letter are not statistically different; groups with different letters have a *p*-value < 0.05. Red lines represent medians. Dashed lines within the violin plots represent quartiles. (**B**) Kaplan–Meier plots of the overall survival of breast cancer patients whose tumors had high (top 5%, *n* = 54) or low (bottom 95%, *n* = 1028) expression of the FDS. Data were obtained from the TCGA Pan-Cancer Atlas. Log-rank test, *p* < 0.05. (**C**) GSEA plot and (**D**) heatmap of genes in FDS evaluated in RNA-seq data from MDA-MB-231 orthotopic xenografts treated with vehicle or rapamycin for three days.

We used the FDS to assess the extent of baseline FAK activation in breast cancers in the TCGA, PanCancer Atlas dataset. Tumors were classified by PAM50 subtypes with TNBCs comprising the majority of the basal breast cancer subtype [50]. The expression of the FAK-dependent gene signature was higher in the highly aggressive breast cancer subtypes: Basal, HER2, and Luminal B compared to Luminal A and Normal-like tumors (Figure 4A). Furthermore, we found that the FDS was significantly associated with worse overall survival from all breast cancers (Figure 4B).

We also found that the FDS was significantly enriched in MDA-MB-231 xenografted tumors in mice treated with rapamycin compared to vehicle control (Figure 4C, NES = 1.4, FDR = 0.106). Of the 20 genes within the FDS that were significantly altered in tumors treated with rapamycin, the majority were up-regulated (Figure 4D). These data indicate that mTORC1 inhibition leads to the up-regulation of genes that are dependent on FAK signaling, suggesting that these tumors may have a dependency on FAK that is revealed when they are exposed to mTORC1i.

### 3.5. FAK Blockade Conveys Sensitivity to mTORC1 Inhibition in Resistant/Moderately Resistant Tumors

**Figure 5:**
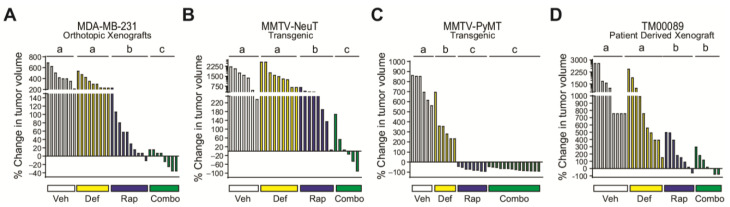
FAK blockade restores sensitivity to mTORC1 inhibition in resistant tumors. (**A**) Waterfall plot of MDA-MB-231 tumors in NSG mice treated with vehicle (Veh), rapamycin (Rap), defactinib (Def), or the combination (Combo, 1:1 ratio) for 14 days. Calculated percent change was based on the tumor size at day 14 compared to the initial tumor size at day 0. Each bar represents one tumor; 5 mice/group with 1–2 tumors/mouse. Groups with the same letter are not statistically different; groups with different letters are statistically different at *p* < 0.05. (**B**) Waterfall plot of MMTV-NeuT transgenic mouse tumors transplanted into FVB/N female mammary fat pads and treated as in (**A**) for 21 days. (**C**) Waterfall plot of MMTV-PyMT transgenic mouse tumors transplanted into FVB/N female mice and treated as in (**A**) for 15 days. (**D**) Waterfall plot of TM00089 PDX tumors in NSG mice treated as in (**A**) for 30 days. a,b,c,d: groups with the same letter are not statistically different; groups with different letters have a *p*-value < 0.05.

Given the up-regulation of the FDS in response to mTORC1 inhibition, we postulated that the efficacy of mTORC1i may be enhanced by additionally targeting FAK. Indeed, prior reports have indicated that the inhibition of mTORC1 and FAK is synergistic in vitro. However, we found that FAK inhibition had minimal, if any, impact on rapamycin efficacy in three different breast cancer cell line models, in vitro (Supplementary Figure S5). Given the ability of mTORC1 to modulate the ECM (Figure 1), we postulated that the impact of FAK inhibition may require signaling that stems from the tumor microenvironment and is absent in the cell culture setting. Thus, to evaluate the impact of FAK inhibition on mTORC1i efficacy in a more relevant biological setting that includes the tumor microenvironment, we transitioned to assessing the impact of adding FAK inhibitors to mTORC1 inhibition using in vivo models. We used two mouse models that have varying levels of intrinsic resistance to rapamycin. These included MDA-MB-231 orthotopic xenografts, as well as a syngeneic, orthotopic transplantable tumor model derived from the MMTV-NeuT mouse model of breast cancer [34,51]. Mice with palpable tumors were treated with vehicle, rapamycin (mTORC1i), defactinib (FAK inhibitor), or the combination, and tumor response was assessed (Figure 5A,B). In these two models, defactinib alone failed to impact tumor growth, while rapamycin either modestly (MDA-MB-231) or minimally (MMTV-NeuT) inhibited growth. Notably, MDA-MB-231 tumors were more responsive than the in vitro cultured cell line (Figure 1A), highlighting the importance of the in vivo setting in defining the extent of the mTORC1i response. In a striking contrast to each drug alone, adding defactinib to rapamycin resulted in considerable suppression of tumor growth and, in multiple cases, induced tumor regression. We then evaluated the impact of defactinib on rapamycin efficacy in two models that are more sensitive to mTORC1 inhibition (Figure 5C,D). These included a syngeneic and orthotopic transplantable tumor model derived from MMTV-PyMT mice or an orthotopic patient-derived xenograft (PDX, TM00089) [52]. In these models, rapamycin alone was much more effective than what was observed in the MDA-MB-231 or MMTV-NeuT models, while defactinib either partially inhibited growth (MMTV-PyMT) or had no impact when used as a single agent. Most importantly, in contrast to the rapamycin-resistant models, defactinib was unable to improve the response to mTORC1 inhibition. In all models, overt toxicity of the single agents and the combination, as assessed by mouse weights, was not observed (Supplementary Figure S6). Notably, mice treated with rapamycin exhibit a highly variable response, which is concordant with prior observations [34,53]. These data indicate that, for tumors that are intrinsically sensitive to rapamycin, FAK signaling does not facilitate their survival and growth. However, in resistant/moderately resistant tumors, mTORC1 inhibition leads to a collateral dependency on FAK. These data further underscore the role of mTORC1 in controlling the ECM, providing a mechanism for alternative growth signaling through FAK.

## 4. Discussion

**Figure 6:**
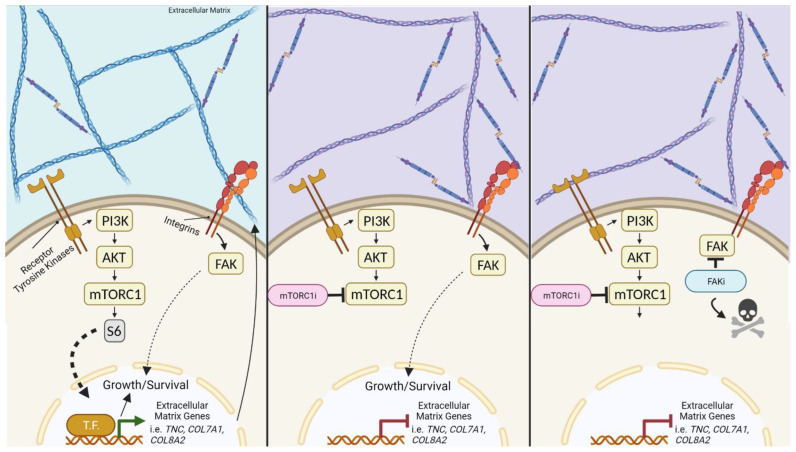
Targeting FAK blocks growth dependence on extracellular matrix following mTORC1 inhibition. mTORC1 inhibition initiates a transcriptional reprogramming of extracellular matrix genes that drives ECM remodeling to maintain growth. The inhibition of FAK abolishes the ability of cells to utilize the ECM as a collateral growth-stimulating pathway. Abbreviations: mTORC1i, mTORC1 inhibition; FAKi, focal adhesion kinase inhibition; T.F., transcription factor.

The mTORC1i rapamycin and its analogs (rapalogs) have been extensively investigated as anti-cancer agents. While rapalogs are approved for treating patients with kidney cancer, pancreatic neuroendocrine tumors, renal cell carcinoma, and luminal breast cancer, success is limited in other cancer types due to intrinsic and adaptive resistance mechanisms that prevent the full efficacy of mTORC1i [9,54,55]. Moreover, for cancers wherein rapalogs are a standard of care, tumors that do respond often develop resistance, as is frequently observed in patients with breast cancer. In this report, we describe a collateral dependency on FAK that occurs in tumors that are resistant to mTORC1i (Figure 6). We discovered that resistance to growth suppression by mTORC1i is associated with extensive rapamycin-induced changes in the expression of genes responsible for forming and remodeling the ECM. This is consistent with studies outside the context of cancer demonstrating changes in collagen expression in response to rapamycin [56,57]. Since FAK is an integrator of ECM signaling that is readily targetable, this implicates FAK as a potential vulnerability that could be leveraged to overcome resistance to mTORC1i. The connection between resistance and the ECM is further supported by the fact that the combined efficacy of mTORC1 and FAK inhibition was only observed in vivo where the ECM is an integral component of the tumor cell microenvironment.

mTOR signaling in breast cancer is active and driven by multiple genetic aberrations within the PAM pathway [3]. Rapamycin inhibits mTORC1, which decreases the activity of p70S6kinase, a regulator of various cancer hallmarks such as survival, DNA damage, and protein synthesis (through S6 activity) [58]. In the context of short-term treatment, the negative regulation of S6 to mTORC2 is relieved; this results in a rebound activation of AKT mediated by mTORC2 [59]. It is unknown in the short-term studies presented herein if mTORC2 is active and may drive the changes in the ECM transcriptional program. It has also been reported that long-term rapamycin exposure indirectly inhibits mTORC2 [59]. It is possible that, in our long-term in vivo studies, mTORC2 is also inhibited. This further suggests that the efficacy of dual mTORC1/mTORC2 inhibitors may also benefit from blocking FAK activity. Indeed, melanoma cells treated with Torin1, an mTOR kinase inhibitor, in combination with FAK inhibitors had a further decrease in migration and invasion in vitro compared to single agents [27]. Together, these studies demonstrate that the impact of mTOR inhibitors can be potentiated by FAK inhibition.

Everolimus (RAD001), a rapamycin analogue, is approved as a second-line therapy for metastatic HR+/HER2- (luminal) breast cancer patients. The activation of the PAM pathway mediates endocrine resistance through intrinsic, adaptive, or acquired mechanisms [60,61]. Additionally, a more recent phase 2 clinical trial demonstrated that everolimus, in addition to letrozole, increases progression-free survival by 5.5 months in women who progressed on selective estrogen receptor modulators, SERMs [62]. However, resistance to mTORC1 inhibition is common, underscoring the need to identify mechanisms of resistance that could lead to the discovery of novel combination therapies that are more efficacious. In this regard, low levels of phosphorylated AKT and S6, an increased EMT signature, and low GSK3α gene expression are associated with resistance to mTORC1i in cell lines [31,32,63]; however, to date, these remain undruggable targets for improving mTORC1i response. PI3KCA mutation status has also been proposed to be a predictor of mTORC1i response in vitro. However, the TAMRAD (tamoxifen + everolimus) and BOLERO-2 (aromatase + everolimus) clinical trials revealed that PI3KCA mutation was not predictive of everolimus response [64,65,66]. As an alternative approach to discovering mechanisms of resistance to mTORC1i, we focused on a breast cancer subtype (TNBC) where intrinsic in vitro resistance is well-established. This approach revealed that the ECM and FAK are novel mediators of response that could be leveraged to improve the efficacy of the prototypical mTORC1i, rapamycin.

The ability of the extracellular matrix to control drug responsiveness has previously been reported within the context of ECM stiffness. Increasing rigidity promotes tumor formation and can induce resistance to therapies targeting mTORC1, HER2, and/or PIK3CA [67,68,69]. In addition, Joyce et al. reported that the placement of TNBC MDA-MB-231 cells into a hydrogel system with increasing stiffness induced resistance to the chemotherapeutic agent doxorubicin [70]. In contrast, the response of MCF7 cells (Luminal A) to doxorubicin was unaffected by changes in the ECM. This study suggests that different breast cancer cells respond to the ECM in distinct ways and that this could control tumor phenotypes, such as therapeutic response. This is further underscored by results presented herein, where differences in the efficacy of mTORC1i were associated with ECM and FAK signaling. While resistant/moderately resistant tumors were sustained and dependent on ECM/FAK signaling, tumors that were highly responsive to mTORC1 blockade failed to activate this pathway, making them particularly vulnerable to the loss of mTORC1 signaling. Importantly, the dependency of tumor cells on FAK signaling following mTORC1 blockade was only revealed using in vivo models of rapamycin-resistant tumors, indicating that drug resistance extends beyond tumor-cell-intrinsic pathways of adaptive or acquired resistance to include the ECM. Together, these data support the concept that inhibiting mTORC1 reveals a collateral sensitivity to signaling from the ECM to tumor cells. Future studies involving genomic or proteomic analyses of pre- and post-treatment tumor biopsies from mTORC1i-sensitive and -resistant tumors may reveal key ECM-related components that can predict drug response. Such studies should reveal biomarkers that can prospectively identify which cancers will benefit from the additional targeting of FAK in the context of mTORC1i resistance.

As indicated above, the discovery that FAK suppression potentiated response to mTORCi was only uncovered when utilizing mouse models of breast cancer and drug synergy was not detected in vitro using extensive Chou–Talalay analyses (Supplementary Figure S3 and data not shown) [71,72]. This finding underscores the importance of using in vivo systems to reveal mechanisms of resistance and assess synergies. Indeed, the in vivo studies mentioned above demonstrating a role for ECM stiffness in controlling response to mTORC1i, combined with the data presented herein, suggest that directly targeting the ECM may enhance the efficacy of targeted therapies. However, ECM rigidity may not be the only factor mediating response to the combined inhibitors in vivo. Additional factors that are modulated by mTORC1i that can also control tumor growth include immune cells, angiogenesis, and cancer-associated fibroblasts (CAFS). Immunological response is unlikely to play a role in the synergy between FAK and mTORC1i because the studies presented here used both immune-compromised and intact hosts [NSG (MDA-MB-231 and TM00089 xenografts) and FVB/N (MMTV-NeuT and MMTV-PyMT syngeneic grafts)] and synergy was independent of immune status. Alternatively, angiogenesis or CAFs could be key targets of the drug combination. The mTORC1i, everolimus and temsirolimus, are antiangiogenic in various tumor models [73]. In addition, FAK signaling in endothelial cells regulates tumor angiogenesis and vascular permeability [74,75,76]. Thus, the addition of FAK inhibitors to mTORC1i may additionally decrease tumor vascularization. CAFs can secrete cytokines, growth factors, and promote ECM remodeling that impacts tumor growth [77]. In response to rapamycin, the expression of various cytokines by fibroblasts is reduced, and this decreases the activation of the PAM pathway in tumor cells [78,79]. Furthermore, an inhibitor of the mTOR/4E-BP1 pathway blocked the protein expression of a CAF chemoresistance secretome in pancreatic cancer [80]. Blocking FAK activity can also decrease ECM production by CAFs that have intrinsically high FAK activity [81]. In breast cancer, genetic disruption of FAK in fibroblasts decreases metastasis in vivo with no impact on primary tumor growth [82]. In contrast, Demircioglu and colleagues recently reported that disrupting FAK in CAF subpopulations causes an increase in cell glycolysis, chemokine production, and tumor growth [83]. In the studies presented here, it is notable that single-agent FAK inhibition only impacted primary tumor growth in one of the four models tested (MMTV-PyMT). It is plausible that the combination of FAK and mTORC1 inhibitors may both potentiate a decrease of CAF response that together impacts primary tumor growth. It is also important to consider that other factors may also contribute to the response to the combined inhibition of FAK and mTORC1. These include the potential of hypoxia, other cell types (i.e., adipocytes and myoepithelial cells), and soluble factors (i.e., matrix remodeling enzymes, cytokines, and growth factors) to modulate the ECM [84].. Discerning the impact of mTORC1 and FAK inhibition on the complex interplay of these factors and their impact on in vivo tumor growth are warranted to discern the potential mechanisms of synergy.

## 5. Conclusions

In summary, tumors that are resistant to mTORC1i rely on FAK activity as an alternative pathway for growth and viability, whereas tumors that are sensitive to mTORC1 blockade fail to utilize this pathway. FAK signaling only becomes essential for growth and viability when mTORC1 is inactivated, indicating that FAK is a collateral sensitivity of mTORC1i resistance. These studies provide a rationale for the future assessment of the efficacy of FAK inhibitors in patients with rapalog-resistant breast cancers.

## Data Availability

Raw data from the RNA-seq can be found under PRJNA830908 (release on 1 May 2023).

## Supplementary Materials

The following supporting information can be downloaded at: https://www.mdpi.com/article/10.3390/cancers14143374/s1, Supplementary Figure S1: Confirmation of mTORC1 inhibition and principal components analysis of MDA-MB-231 samples analyzed by RNA-seq. Supplementary Figure S2: Total protein of breast cancer cell lines. Supplementary Figure S3: Quantitation of FAK Y397 intensity in breast cancer patient samples. Supplementary Figure S4: Development of a FAK-dependent signature (FDS). Supplementary Figure S5: Defactinib fails to potentiate rapamycin efficacy in vitro. Supplementary Figure S6: The combination of rapamycin and defactinib does not induce overt in vivo toxicity. Supplementary Figure S7: Original western blots of mouse tumors. Supplementary Figure S8: Original western blots of breast cancer cell lines. Supplementary Table S1: Gene expression changes following mTORC1 inhibition in MDA-MB-231 xenografts. Supplementary Table S2: Reactome pathways of the top and bottom 10% of all DEG correlated to S6 S235/236. Supplementary Table S3: Reactome pathways of the top and bottom 10% of all DEG correlated to 4E-BP1. Supplementary Table S4: Reactome pathways of the top and bottom 10% of all DEG correlated to 4E-BP1 S65. Supplementary Table S5: Reactome pathways of the top and bottom 10% of all DEG correlated to p70S6K. Supplementary Table S6: Reactome pathways of the top and bottom 10% of all DEG correlated to p70S6K T389.

## References

[1] Lee J.J., Loh K., Yap Y.S. (2015). PI3K/Akt/mTOR inhibitors in breast cancer. Cancer Biol. Med..

[2] Guerrero-Zotano A., Mayer I.A., Arteaga C.L. (2016). PI3K/AKT/mTOR: Role in breast cancer progression, drug resistance, and treatment. Cancer Metastasis Rev..

[3] Cancer Genome Atlas N. (2012). Comprehensive molecular portraits of human breast tumours. Nature.

[4] Mayer I.A., Arteaga C.L. (2016). The PI3K/AKT Pathway as a Target for Cancer Treatment. Annu. Rev. Med..

[5] Pernas S., Tolaney S.M. (2019). HER2-positive breast cancer: New therapeutic frontiers and overcoming resistance. Ther. Adv. Med. Oncol..

[6] Paplomata E., O’Regan R. (2014). The PI3K/AKT/mTOR pathway in breast cancer: Targets, trials and biomarkers. Ther. Adv. Med. Oncol..

[7] Markham A. (2019). Alpelisib: First Global Approval. Drugs.

[8] Andre F., Ciruelos E., Rubovszky G., Campone M., Loibl S., Rugo H.S., Iwata H., Conte P., Mayer I.A., Kaufman B. (2019). Alpelisib for PIK3CA-Mutated, Hormone Receptor-Positive Advanced Breast Cancer. N. Engl. J. Med..

[9] Porta C., Paglino C., Mosca A. (2014). Targeting PI3K/Akt/mTOR Signaling in Cancer. Front. Oncol..

[10] O’Reilly K.E., Rojo F., She Q.B., Solit D., Mills G.B., Smith D., Lane H., Hofmann F., Hicklin D.J., Ludwig D.L. (2006). mTOR inhibition induces upstream receptor tyrosine kinase signaling and activates Akt. Cancer Res..

[11] Rodrik-Outmezguine V.S., Chandarlapaty S., Pagano N.C., Poulikakos P.I., Scaltriti M., Moskatel E., Baselga J., Guichard S., Rosen N. (2011). mTOR kinase inhibition causes feedback-dependent biphasic regulation of AKT signaling. Cancer Discov..

[12] Miller S.M., Goulet D.R., Johnson G.L. (2017). Targeting the Breast Cancer Kinome. J. Cell Physiol..

[13] Pluchino K.M., Hall M.D., Goldsborough A.S., Callaghan R., Gottesman M.M. (2012). Collateral sensitivity as a strategy against cancer multidrug resistance. Drug Resist. Updat..

[14] Efferth T., Saeed M.E.M., Kadioglu O., Seo E.J., Shirooie S., Mbaveng A.T., Nabavi S.M., Kuete V. (2020). Collateral sensitivity of natural products in drug-resistant cancer cells. Biotechnol. Adv..

[15] Mueller H.S., Fowler C.E., Dalin S., Moiso E., Udomlumleart T., Garg S., Hemann M.T., Lees J.A. (2021). Acquired resistance to PRMT5 inhibition induces concomitant collateral sensitivity to paclitaxel. Proc. Natl. Acad. Sci. USA.

[16] Koh S.-B., Ross K., Isakoff S.J., Melkonjan N., He L., Matissek K.J., Schultz A., Mayer E.L., Traina T.A., Carey L.A. (2021). RASAL2 Confers Collateral MEK/EGFR Dependency in Chemoresistant Triple-Negative Breast Cancer. Clin. Cancer Res..

[17] Guo W., Giancotti F.G. (2004). Integrin signalling during tumour progression. Nat. Rev. Mol. Cell Biol..

[18] Insua-Rodríguez J., Oskarsson T. (2016). The extracellular matrix in breast cancer. Adv. Drug Deliv. Rev..

[19] Sulzmaier F.J., Jean C., Schlaepfer D.D. (2014). FAK in cancer: Mechanistic findings and clinical applications. Nat. Cancer.

[20] Lee B.Y., Timpson P., Horvath L.G., Daly R.J. (2015). FAK signaling in human cancer as a target for therapeutics. Pharmacol. Ther..

[21] Schwill M., Tamaskovic R., Gajadhar A.S., Kast F., White F.M., Plückthun A. (2019). Systemic analysis of tyrosine kinase signaling reveals a common adaptive response program in a HER2-positive breast cancer. Sci. Signal..

[22] Stuhlmiller T.J., Miller S.M., Zawistowski J.S., Nakamura K., Beltran A.S., Duncan J.S., Angus S.P., Collins K.A., Granger D.A., Reuther R.A. (2015). Inhibition of Lapatinib-Induced Kinome Reprogramming in ERBB2-Positive Breast Cancer by Targeting BET Family Bromodomains. Cell Rep..

[23] Huang C., Park C.C., Hilsenbeck S.G., Ward R., Rimawi M.F., Wang Y.C., Shou J., Bissell M.J., Osborne C.K., Schiff R. (2011). beta1 integrin mediates an alternative survival pathway in breast cancer cells resistant to lapatinib. Breast Cancer Res..

[24] Jin M.H., Nam A.-R., Park J.E., Bang J.-H., Bang Y.-J., Oh D.-Y. (2017). Resistance Mechanism against Trastuzumab in HER2-Positive Cancer Cells and Its Negation by Src Inhibition. Mol. Cancer Ther..

[25] Kessler B.E., Mishall K.M., Kellett M.D., Clark E.G., Pugazhenthi U., Pozdeyev N., Kim J., Tan A.C., Schweppe R.E. (2018). Resistance to Src inhibition alters the BRAF-mutant tumor secretome to promote an invasive phenotype and therapeutic escape through a FAK>p130Cas>c-Jun signaling axis. Oncogene.

[26] Ichihara E., Westover D., Meador C.B., Yan Y., Bauer J.A., Lu P., Ye F., Kulick A., de Stanchina E., McEwen R. (2017). SFK/FAK Signaling Attenuates Osimertinib Efficacy in Both Drug-Sensitive and Drug-Resistant Models of EGFR-Mutant Lung Cancer. Cancer Res..

[27] Yoon S.O., Shin S., Karreth F.A., Buel G.R., Jedrychowski M.P., Plas D.R., Dedhar S., Gygi S.P., Roux P.P., Dephoure N. (2017). Focal Adhesion- and IGF1R-Dependent Survival and Migratory Pathways Mediate Tumor Resistance to mTORC1/2 Inhibition. Mol. Cell.

[28] Francois R.A., Maeng K., Nawab A., Kaye F.J., Hochwald S.N., Zajac-Kaye M. (2015). Targeting Focal Adhesion Kinase and Resistance to mTOR Inhibition in Pancreatic Neuroendocrine Tumors. J. Natl. Cancer Inst..

[29] Shi P.-J., Xu L.-H., Lin K.-Y., Weng W.-J., Fang J.-P. (2016). Synergism between the mTOR inhibitor rapamycin and FAK down-regulation in the treatment of acute lymphoblastic leukemia. J. Hematol. Oncol..

[30] You D., Xin J., Volk A., Wei W., Schmidt R., Scurti G., Nand S., Breuer E.-K., Kuo P.C., Breslin P. (2015). FAK Mediates a Compensatory Survival Signal Parallel to PI3K-AKT in PTEN-Null T-ALL Cells. Cell Rep..

[31] Noh W.-C., Mondesire W.H., Peng J., Jian W., Zhang H., Dong J., Mills G.B., Hung M.-C., Meric-Bernstam F. (2004). Determinants of Rapamycin Sensitivity in Breast Cancer Cells. Clin. Cancer Res..

[32] Hurvitz S.A., Kalous O., Conklin D., Desai A.J., Dering J., Anderson L., O’Brien N.A., Kolarova T., Finn R.S., Linnartz R. (2015). In vitro activity of the mTOR inhibitor everolimus, in a large panel of breast cancer cell lines and analysis for predictors of response. Breast Cancer Res. Treat..

[33] Yoon H., Dehart J.P., Murphy J.M., Lim S.T. (2015). Understanding the roles of FAK in cancer: Inhibitors, genetic models, and new insights. J. Histochem. Cytochem..

[34] Yori J.L., Lozada K.L., Seachrist D.D., Mosley J.D., Abdul-Karim F.W., Booth C.N., Flask C.A., Keri R.A. (2014). Combined SFK/mTOR Inhibition Prevents Rapamycin-Induced Feedback Activation of AKT and Elicits Efficient Tumor Regression. Cancer Res..

[35] Sizemore S.T., Sizemore G.M., Booth C.N., Thompson C.L., Silverman P., Bebek G., Abdul-Karim F.W., Avril S., Keri R.A. (2014). Hypomethylation of the MMP7 promoter and increased expression of MMP7 distinguishes the basal-like breast cancer subtype from other triple-negative tumors. Breast Cancer Res. Treat.

[36] Kamburov A., Pentchev K., Galicka H., Wierling C., Lehrach H., Herwig R. (2010). ConsensusPathDB: Toward a more complete picture of cell biology. Nucleic Acids Res..

[37] Kamburov A., Wierling C., Lehrach H., Herwig R. (2009). ConsensusPathDB--a database for integrating human functional interaction networks. Nucleic Acids Res..

[38] Subramanian A., Tamayo P., Mootha V.K., Mukherjee S., Ebert B.L., Gillette M.A., Paulovich A., Pomeroy S.L., Golub T.R., Lander E.S. (2005). Gene set enrichment analysis: A knowledge-based approach for interpreting genome-wide expression profiles. Proc. Natl. Acad. Sci. USA.

[39] Mootha V.K., Lindgren C.M., Eriksson K.F., Subramanian A., Sihag S., Lehar J., Puigserver P., Carlsson E., Ridderstrale M., Laurila E. (2003). PGC-1alpha-responsive genes involved in oxidative phosphorylation are coordinately downregulated in human diabetes. Nat. Genet..

[40] Ciriello G., Gatza M.L., Beck A.H., Wilkerson M.D., Rhie S.K., Pastore A., Zhang H., McLellan M., Yau C., Kandoth C. (2015). Comprehensive Molecular Portraits of Invasive Lobular Breast Cancer. Cell.

[41] Gao J., Aksoy B.A., Dogrusoz U., Dresdner G., Gross B.E., Sumer S.O., Sun Y., Jacobsen A., Sinha R., Larsson E. (2013). Integrative Analysis of Complex Cancer Genomics and Clinical Profiles Using the cBioPortal. Sci. Signal..

[42] Cerami E., Gao J., Dogrusoz U., Gross B.E., Sumer S.O., Aksoy B.A., Jacobsen A., Byrne C.J., Heuer M.L., Larsson E. (2012). The cBio cancer genomics portal: An open platform for exploring multidimensional cancer genomics data. Cancer Discov..

[43] Dai X., Cheng H., Bai Z., Li J. (2017). Breast Cancer Cell Line Classification and Its Relevance with Breast Tumor Subtyping. J. Cancer.

[44] Vranic S., Gatalica Z., Wang Z.-Y. (2011). Update on the molecular profile of the MDA-MB-453 cell line as a model for apocrine breast carcinoma studies. Oncol. Lett..

[45] Prat A., Parker J.S., Karginova O., Fan C., Livasy C., Herschkowitz J.I., He X., Perou C.M. (2010). Phenotypic and molecular characterization of the claudin-low intrinsic subtype of breast cancer. Breast Cancer Res..

[46] Iorns E., Drews-Elger K., Ward T.M., Dean S., Clarke J., Berry D., El Ashry D., Lippman M. (2012). A New Mouse Model for the Study of Human Breast Cancer Metastasis. PLoS ONE.

[47] Hamidi H., Ivaska J. (2018). Every step of the way: Integrins in cancer progression and metastasis. Nat. Rev. Cancer.

[48] McLean G.W., Carragher N.O., Avizienyte E., Evans J., Brunton V.G., Frame M.C. (2005). The role of focal-adhesion kinase in cancer-A new therapeutic opportunity. Nat. Rev. Cancer.

[49] Shen M., Jiang Y.Z., Wei Y., Ell B., Sheng X., Esposito M., Kang J., Hang X., Zheng H., Rowicki M. (2019). Tinagl1 Suppresses Triple-Negative Breast Cancer Progression and Metastasis by Simultaneously Inhibiting Integrin/FAK and EGFR Signaling. Cancer Cell..

[50] Alluri P., Newman L.A. (2014). Basal-like and triple-negative breast cancers: Searching for positives among many negatives. Surg. Oncol. Clin. N. Am..

[51] Muller W.J., Arteaga C.L., Muthuswamy S.K., Siegel P.M., Webster M.A., Cardiff R.D., Meise K.S., Li F., Halter S.A., Coffey R.J. (1996). Synergistic interaction of the Neu proto-oncogene product and transforming growth factor alpha in the mammary epithelium of transgenic mice. Mol. Cell. Biol..

[52] Guy C.T., Cardiff R.D., Muller W.J. (1992). Induction of mammary tumors by expression of polyomavirus middle T oncogene: A transgenic mouse model for metastatic disease. Mol. Cell. Biol..

[53] Mosley J.D., Poirier J.T., Seachrist D.D., Landis M.D., Keri R.A. (2007). Rapamycin inhibits multiple stages of c-Neu/ErbB2 induced tumor progression in a transgenic mouse model of HER2-positive breast cancer. Mol. Cancer Ther..

[54] Steelman L.S., Martelli A.M., Cocco L., Libra M., Nicoletti F., Abrams S.L., McCubrey J.A. (2016). The therapeutic potential of mTOR inhibitors in breast cancer. Br. J. Clin. Pharmacol..

[55] Lebwohl D., Anak O., Sahmoud T., Klimovsky J., Elmroth I., Haas T., Posluszny J., Saletan S., Berg W. (2013). Development of everolimus, a novel oral mTOR inhibitor, across a spectrum of diseases. Ann. N. Y. Acad. Sci..

[56] Chung C.L., Lawrence I., Hoffman M., Elgindi D., Nadhan K., Potnis M., Jin A., Sershon C., Binnebose R., Lorenzini A. (2019). Topical rapamycin reduces markers of senescence and aging in human skin: An exploratory, prospective, randomized trial. GeroScience.

[57] Shegogue D., Trojanowska M. (2004). Mammalian target of rapamycin positively regulates collagen type I production via a phosphatidylinositol 3-kinase-independent pathway. J. Biol. Chem..

[58] Bahrami B.F., Ataie-Kachoie P., Pourgholami M.H., Morris D.L. (2014). p70 Ribosomal protein S6 kinase (Rps6kb1): An update. J. Clin. Pathol..

[59] Li J., Kim S.G., Blenis J. (2014). Rapamycin: One drug, many effects. Cell. Metab..

[60] Shtivelband M.I. (2013). Everolimus in hormone receptor–positive advanced breast cancer: Targeting receptor-based mechanisms of resistance. Breast.

[61] Schettini F., Buono G., Trivedi M.V., De Placido S., Arpino G., Giuliano M. (2017). PI3K/mTOR Inhibitors in the Treatment of Luminal Breast Cancer. Why, When and to Whom. Breast Care.

[62] Fan Y., Sun T., Shao Z., Zhang Q., Ouyang Q., Tong Z., Wang S., Luo Y., Teng Y., Wang X. (2021). Effectiveness of Adding Everolimus to the First-line Treatment of Advanced Breast Cancer in Premenopausal Women Who Experienced Disease Progression While Receiving Selective Estrogen Receptor Modulators: A Phase 2 Randomized Clinical Trial. JAMA Oncol..

[63] Holder A.M., Akcakanat A., Adkins F., Evans K., Chen H., Wei C., Milton D.R., Li Y., Do K.-A., Janku F. (2015). Epithelial to mesenchymal transition is associated with rapamycin resistance. Oncotarget.

[64] Yi Z., Ma F. (2017). Biomarkers of Everolimus Sensitivity in Hormone Receptor-Positive Breast Cancer. J. Breast Cancer.

[65] Omarini C., Filieri M.E., Bettelli S., Manfredini S., Kaleci S., Caprera C., Nasso C., Barbolini M., Guaitoli G., Moscetti L. (2018). Mutational Profile of Metastatic Breast Cancer Tissue in Patients Treated with Exemestane Plus Everolimus. BioMed Res. Int..

[66] Citi V., Del Re M., Martelli A., Calderone V., Breschi M.C., Danesi R. (2018). Phosphorylation of AKT and ERK1/2 and mutations of PIK3CA and PTEN are predictive of breast cancer cell sensitivity to everolimus in vitro. Cancer Chemother. Pharmacol..

[67] Hanker A.B., Estrada M.V., Bianchini G., Moore P.D., Zhao J., Cheng F., Koch J.P., Gianni L., Tyson D.R., Sanchez V. (2017). Extracellular Matrix/Integrin Signaling Promotes Resistance to Combined Inhibition of HER2 and PI3K in HER2(+) Breast Cancer. Cancer Res..

[68] Weigelt B., Lo A.T., Park C.C., Gray J.W., Bissell M.J. (2009). HER2 signaling pathway activation and response of breast cancer cells to HER2-targeting agents is dependent strongly on the 3D microenvironment. Breast Cancer Res. Treat..

[69] Shea M.P., O’Leary K.A., Wegner K.A., Vezina C.M., Schuler L.A. (2018). High collagen density augments mTOR-dependent cancer stem cells in ERalpha+ mammary carcinomas, and increases mTOR-independent lung metastases. Cancer Lett..

[70] Joyce M.H., Lu C., James E.R., Hegab R., Allen S.C., Suggs L.J., Brock A. (2018). Phenotypic Basis for Matrix Stiffness-Dependent Chemoresistance of Breast Cancer Cells to Doxorubicin. Front. Oncol..

[71] Chou T.C. (2006). Theoretical basis, experimental design, and computerized simulation of synergism and antagonism in drug combination studies. Pharmacol. Rev..

[72] Chou T.C. (2010). Drug combination studies and their synergy quantification using the Chou-Talalay method. Cancer Res..

[73] Lane H.A., Wood J.M., McSheehy P.M., Allegrini P.R., Boulay A., Brueggen J., Littlewood-Evans A., Maira S.M., Martiny-Baron G., Schnell C.R. (2009). mTOR inhibitor RAD001 (everolimus) has antiangiogenic/vascular properties distinct from a VEGFR tyrosine kinase inhibitor. Clin. Cancer Res..

[74] Zhou J., Yi Q., Tang L. (2019). The roles of nuclear focal adhesion kinase (FAK) on Cancer: A focused review. J. Exp. Clin. Cancer Res..

[75] Pedrosa A.-R., Bodrug N., Gomez-Escudero J., Carter E.P., Reynolds L.E., Georgiou P.N., Fernandez I., Lees D.M., Kostourou V., Alexopoulou A.N. (2019). Tumor Angiogenesis Is Differentially Regulated by Phosphorylation of Endothelial Cell Focal Adhesion Kinase Tyrosines-397 and -861. Cancer Res..

[76] Tavora B., Batista S., Reynolds L.E., Jadeja S., Robinson S., Kostourou V., Hart I., Fruttiger M., Parsons M., Hodivala-Dilke K.M. (2010). Endothelial FAK is required for tumour angiogenesis. EMBO Mol. Med..

[77] Conciatori F., Bazzichetto C., Falcone I., Pilotto S., Bria E., Cognetti F., Milella M., Ciuffreda L. (2018). Role of mTOR Signaling in Tumor Microenvironment: An Overview. Int. J. Mol. Sci..

[78] Gillespie Z.E., MacKay K., Sander M., Trost B., Dawicki W., Wickramarathna A., Gordon J., Eramian M., Kill I.R., Bridger J.M. (2015). Rapamycin reduces fibroblast proliferation without causing quiescence and induces STAT5A/B-mediated cytokine production. Nucleus.

[79] Heits N., Heinze T., Bernsmeier A., Kerber J., Hauser C., Becker T., Kalthoff H., Egberts J.H., Braun F. (2016). Influence of mTOR-inhibitors and mycophenolic acid on human cholangiocellular carcinoma and cancer associated fibroblasts. BMC Cancer.

[80] Duluc C., Moatassim-Billah S., Chalabi-Dchar M., Perraud A., Samain R., Breibach F., Gayral M., Cordelier P., Delisle M.B., Bousquet-Dubouch M.P. (2015). Pharmacological targeting of the protein synthesis mTOR/4E-BP1 pathway in cancer-associated fibroblasts abrogates pancreatic tumour chemoresistance. EMBO Mol. Med..

[81] Zaghdoudi S., Decaup E., Belhabib I., Samain R., Cassant-Sourdy S., Rochotte J., Brunel A., Schlaepfer D., Cros J., Neuzillet C. (2020). FAK activity in cancer-associated fibroblasts is a prognostic marker and a druggable key metastatic player in pancreatic cancer. EMBO Mol. Med..

[82] Wu H.J., Hao M., Yeo S.K., Guan J.L. (2020). FAK signaling in cancer-associated fibroblasts promotes breast cancer cell migration and metastasis by exosomal miRNAs-mediated intercellular communication. Oncogene.

[83] Demircioglu F., Wang J., Candido J., Costa A.S.H., Casado P., de Luxan Delgado B., Reynolds L.E., Gomez-Escudero J., Newport E., Rajeeve V. (2020). Cancer associated fibroblast FAK regulates malignant cell metabolism. Nat. Commun..

[84] Li J.J., Tsang J.Y., Tse G.M. (2021). Tumor Microenvironment in Breast Cancer-Updates on Therapeutic Implications and Pathologic Assessment. Cancers.

