# Supplementary material for: Focal Adhesion Kinase Provides a Collateral Vulnerability That Can Be Leveraged to Improve mTORC1 Inhibitor Efficacy
Source: Cancers (Basel). 2022 Jul 11;14(14):3374. doi: 10.3390/cancers14143374 (PMC9323520; doi:10.3390/cancers14143374)
Supplement: Supplementary file 1 [file cancers-14-03374-s001.zip › cancers-1779871-Supplementary Figures .pdf]

Supplementary Materials: Focal Adhesion Kinase Provides a Collateral Vulnerability That Can Be Leveraged to Improve mTORC1 Inhibitor Efficacy

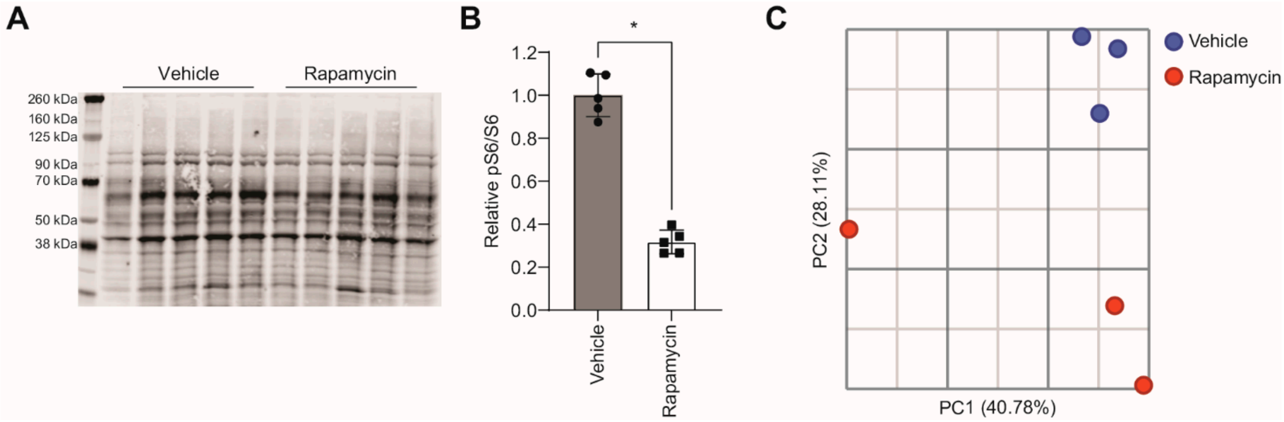

Supplementary Figure S1: Confirmation of mTORC1 inhibition and principal components analysis of MDA-MB-231 samples analyzed by RNA-seq

A. Total protein stained membrane of the blot shown in Figure 1B. On the left, select molecular weights of the protein ladder. **B.** Quantitation of pS6 S235/236 relative to total S6 protein from western blots. *, p-value < 0.05.**B.** PCA analysis of vehicle and rapamycin treatment tumor samples.

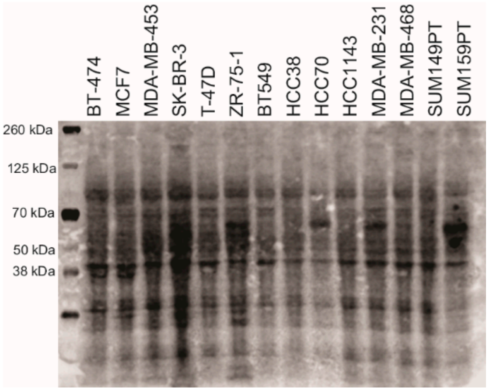

Supplementary Figure S2: Total protein of breast cancer cell lines

Total protein stained membrane of the blot shown in Figure 2D. On the left, select molecular weights of the protein ladder.

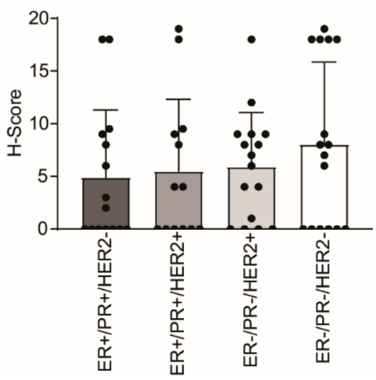

Supplementary Figure S3: Quantitation of FAK Y397 intensity in breast cancer patient samples

H-score of pFAK Y397 stained tumors across breast cancer subtypes.

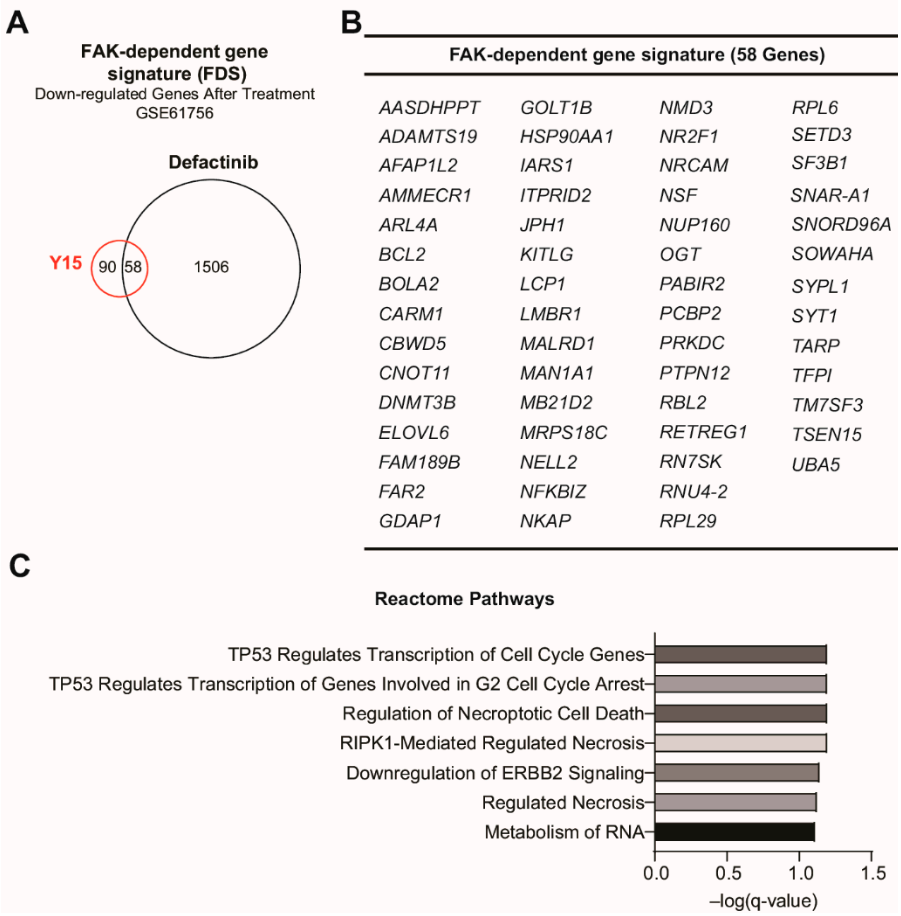

Supplementary Figure S4: Development of a FAK-dependent signature (FDS)

A. Interrogation of the GSE1756 dataset to identify a FAK-dependent signature. Shown is a Venn diagram illustrating the overlap of 58 differentially downregulated genes in SK-BR-3 cells that were treated with either 1μM Y15 (red circle) or PF04554878 (Defactinib, black circle) for 24hrs. Differentially expressed genes (*p*>0.05) were identified using GEO2R for each drug. The overlapping repressed genes comprise the FDS. **B.** Table of the 58 genes comprising the FAK-regulated gene signature. **C.** Reactome pathways that are significantly enriched in the FDS.

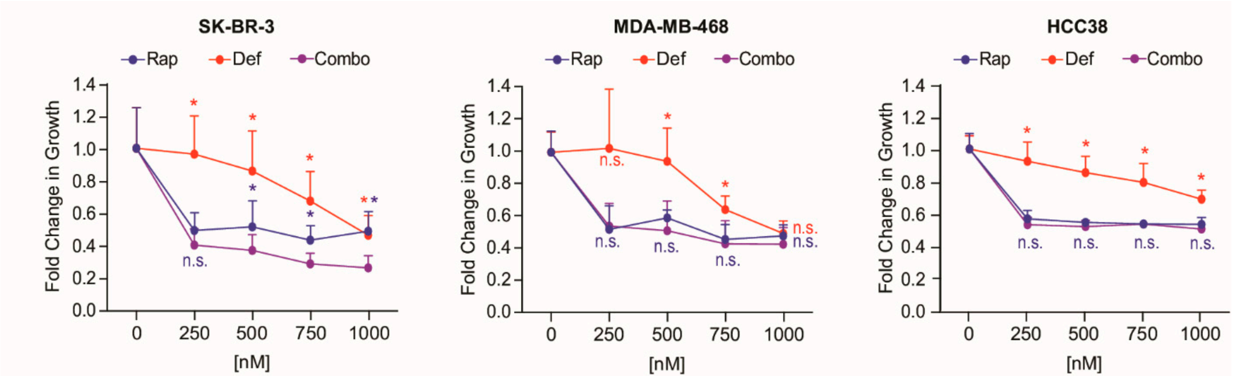

Supplementary Figure S5: Defactinib fails to potentiate rapamycin efficacy *in vitro*

Breast cancer cells were treated with increasing doses of rapamycin (Rap), defactinib (Def), or the combination (Combo) at 1:1 ratios from 250nM to 1uM for 7 days. Cells were stained by crystal violet and quantified by absorbance. *, p-value < 0.05 compared to combination treatment. n.s., non-significant. Colors correspond to the treatment group compared to combination treatment: blue, rapamycin; red, defactinib; purple, combination.

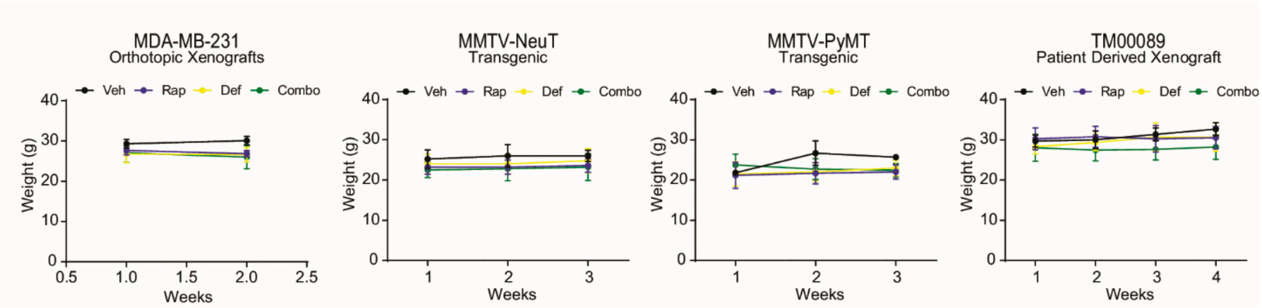

Supplementary Figure S6: The combination of rapamycin and defactinib does not induce overt in vivo toxicity

Mice were treated with vehicle (Veh), rapamycin (Rap), defactinib (Def), or the combination (1:1 ratio). Mouse weights were measured weekly during each treatment paradigm (g, grams). MDA-MB-231 tumors were treated for 14 days. MMTV-NeuT tumors were treated for 21 days. MMTV-PyMT tumors were treated for 15 days. Mice bearing PDX TM00089 were treated for 30 days. blue, rapamycin; red, defactinib; purple, combination.

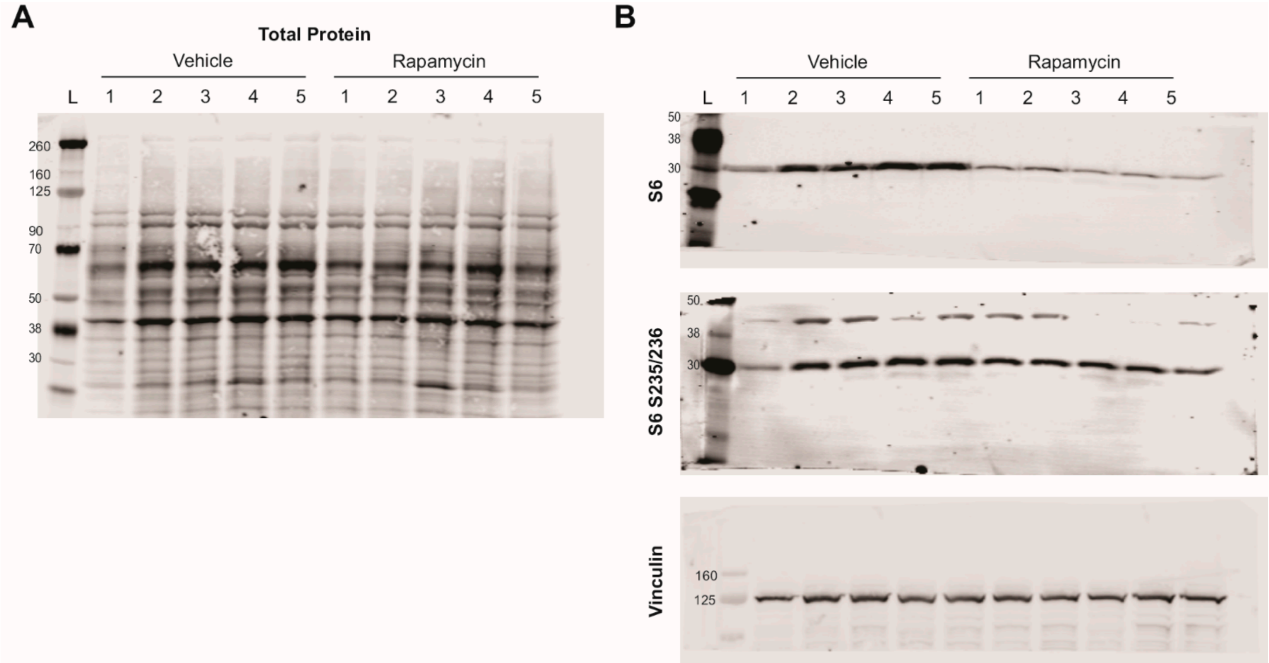

Supplementary Figure S7: Original western blots of mouse tumors

Original western blots of the images presented in Figure 1B and Supplementary Figure 1A. Full blots were first stained and imaged with total protein stain, REVERT. The membrane was then cut at 50kDa. **A.** Total protein stain. L = ladder. Numbers are molecular weights (kDa) of the protein ladder. **B.** Top, S6; Middle, S6 S235/236; Bottom, Vinculin.

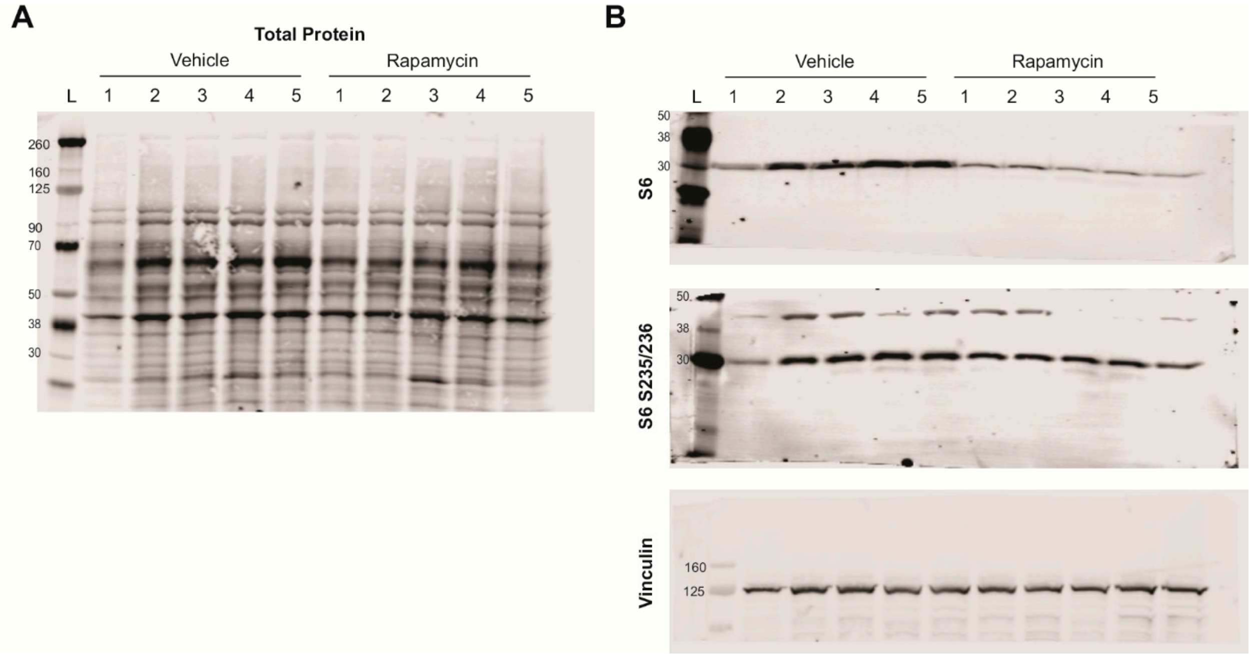

Supplementary Figure S8: Original western blots of breast cancer cell lines

Original western blots of the images presented in Figure 2D and Supplementary Figure 2. Full blots were first stained and imaged with total protein stain, REVERT. The membrane was then cut at 50kDa. **A.** Total protein stain. L = ladder. Numbers are molecular weights (kDa) of the protein ladder. **B.** Top, FAK; Middle, FAK Y397; Bottom, β-Actin.

Supplementary Tables S1–S6 are in separate files

Supplementary Table S1: Gene expression changes following mTORC1 inhibition in MDA-MB-231 xenografts

Supplementary Table S2: Reactome pathways of the top and bottom 10% of all DEG correlated with S6 S235/236

Supplementary Table S3: Reactome pathways of the top and bottom 10% of all DEG correlated with 4E-BP1

Supplementary Table S4: Reactome pathways of the top and bottom 10% of all DEG correlated with 4E-BP1 S65

Supplementary Table S5: Reactome pathways of the top and bottom 10% of all DEG correlated with p70S6K

Supplementary Table S6: Reactome pathways of the top and bottom 10% of all DEG correlated with p70S6K T389
