# Supplementary figures and images for: Focal Adhesion Kinase Provides a Collateral Vulnerability That Can Be Leveraged to Improve mTORC1 Inhibitor Efficacy
Source: Cancers (Basel). 2022 Jul 11;14(14):3374. doi: 10.3390/cancers14143374 (PMC9323520; doi:10.3390/cancers14143374)

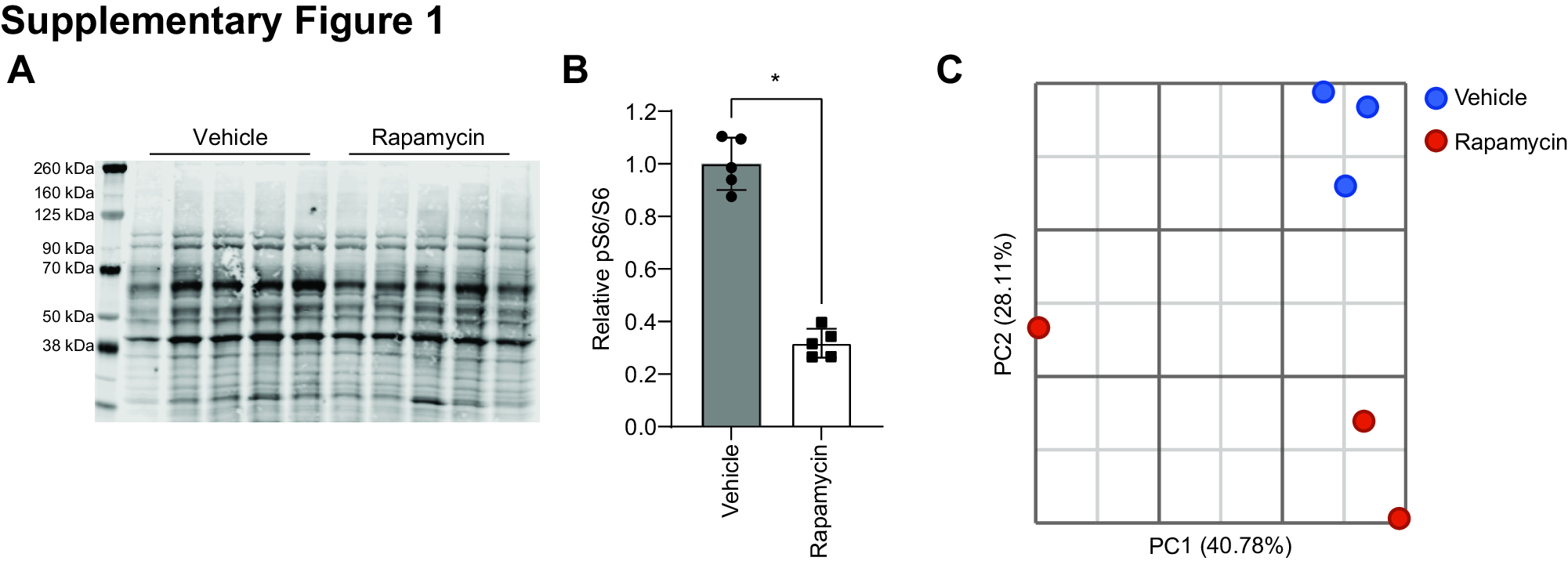

Supplement: Supplementary file 1 [file cancers-14-03374-s001.zip › Supplementary Figure S1.tiff]

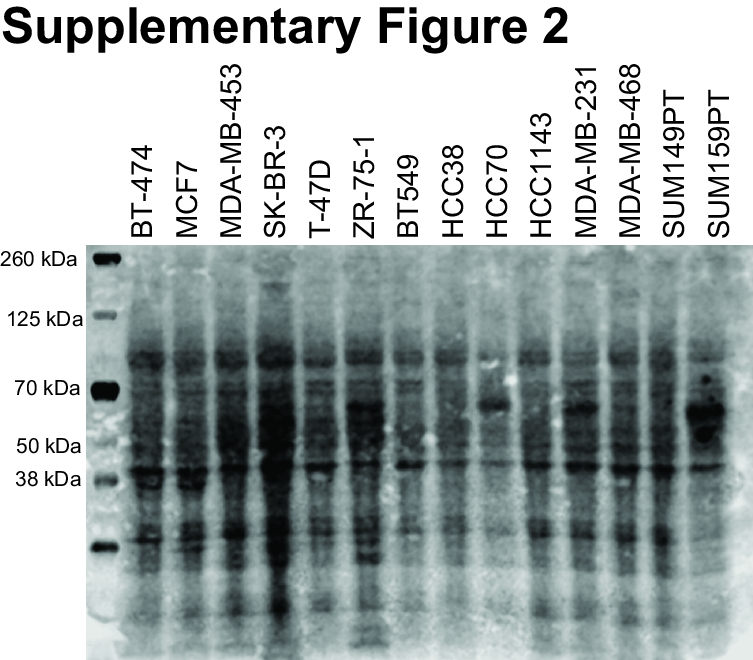

Supplement: Supplementary file 1 [file cancers-14-03374-s001.zip › Supplementary Figure S2.tiff]

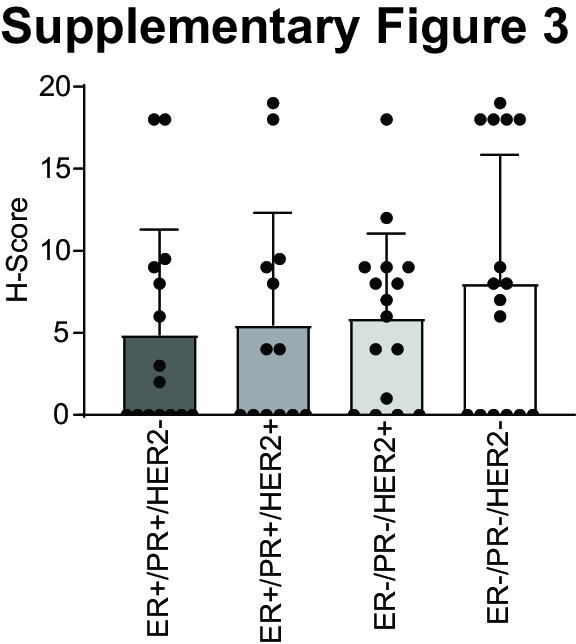

Supplement: Supplementary file 1 [file cancers-14-03374-s001.zip › Supplementary Figure S3.tiff]

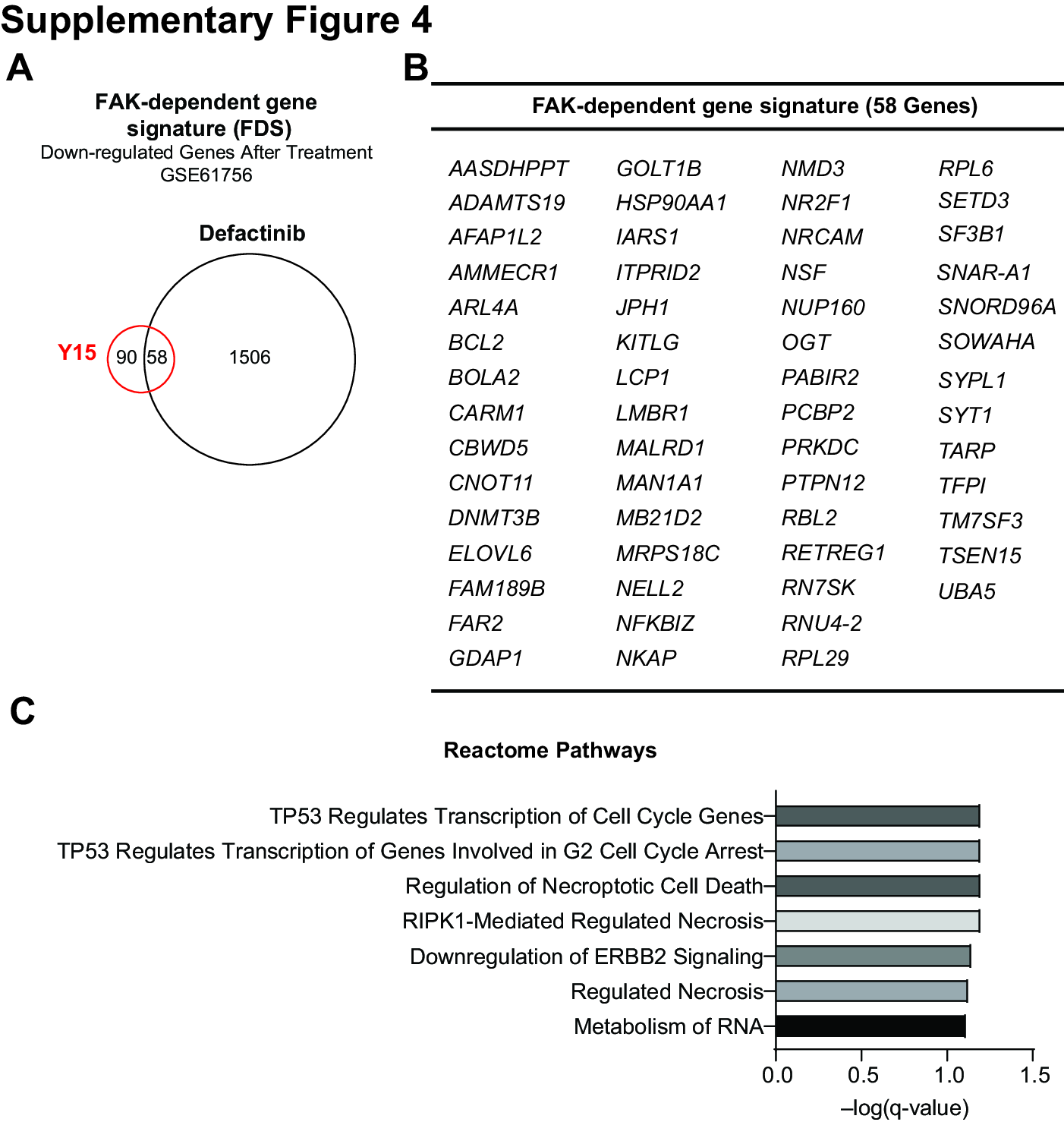

Supplement: Supplementary file 1 [file cancers-14-03374-s001.zip › Supplementary Figure S4.tiff]

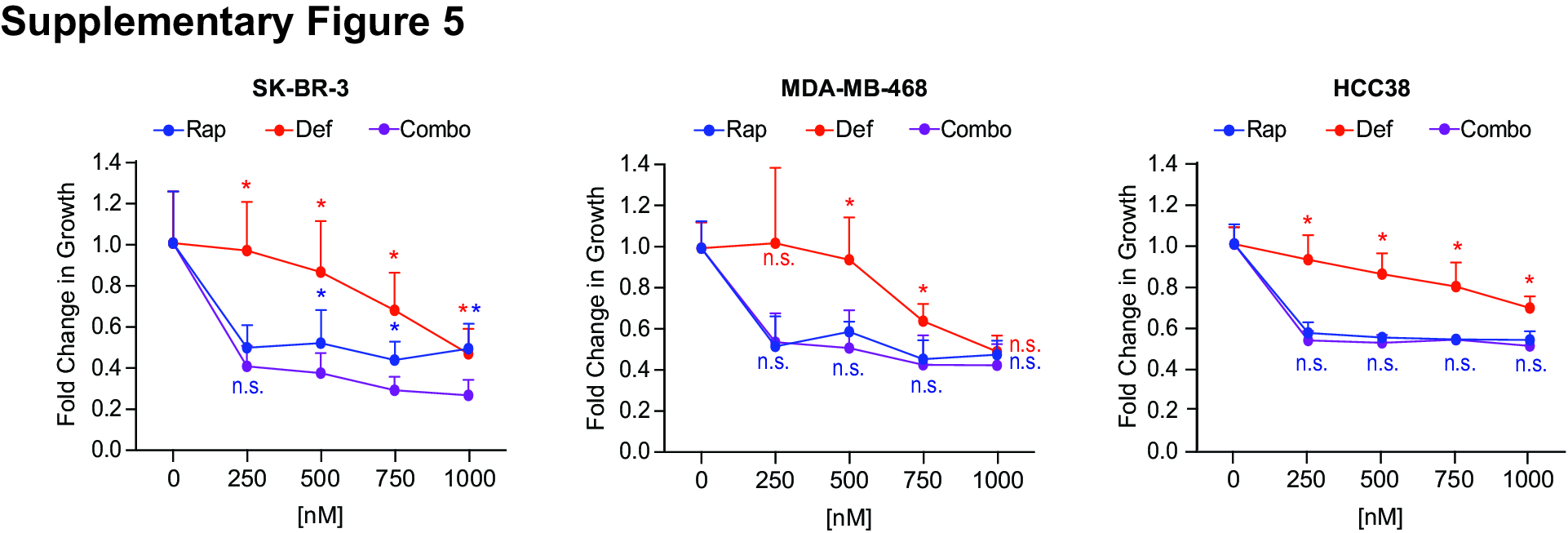

Supplement: Supplementary file 1 [file cancers-14-03374-s001.zip › Supplementary Figure S5.tiff]

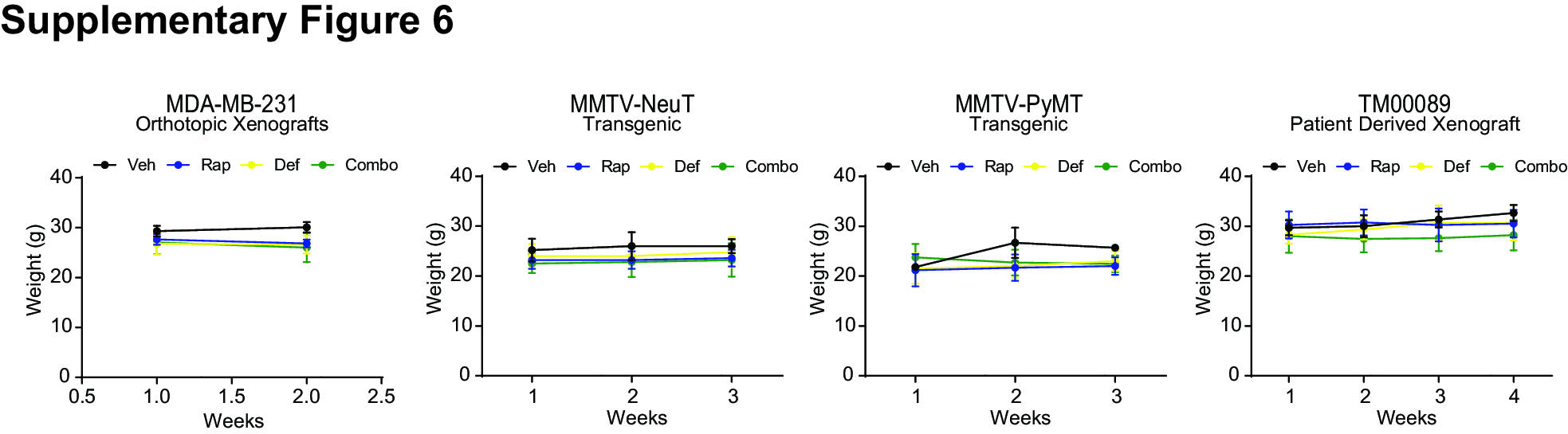

Supplement: Supplementary file 1 [file cancers-14-03374-s001.zip › Supplementary Figure S6.tiff]

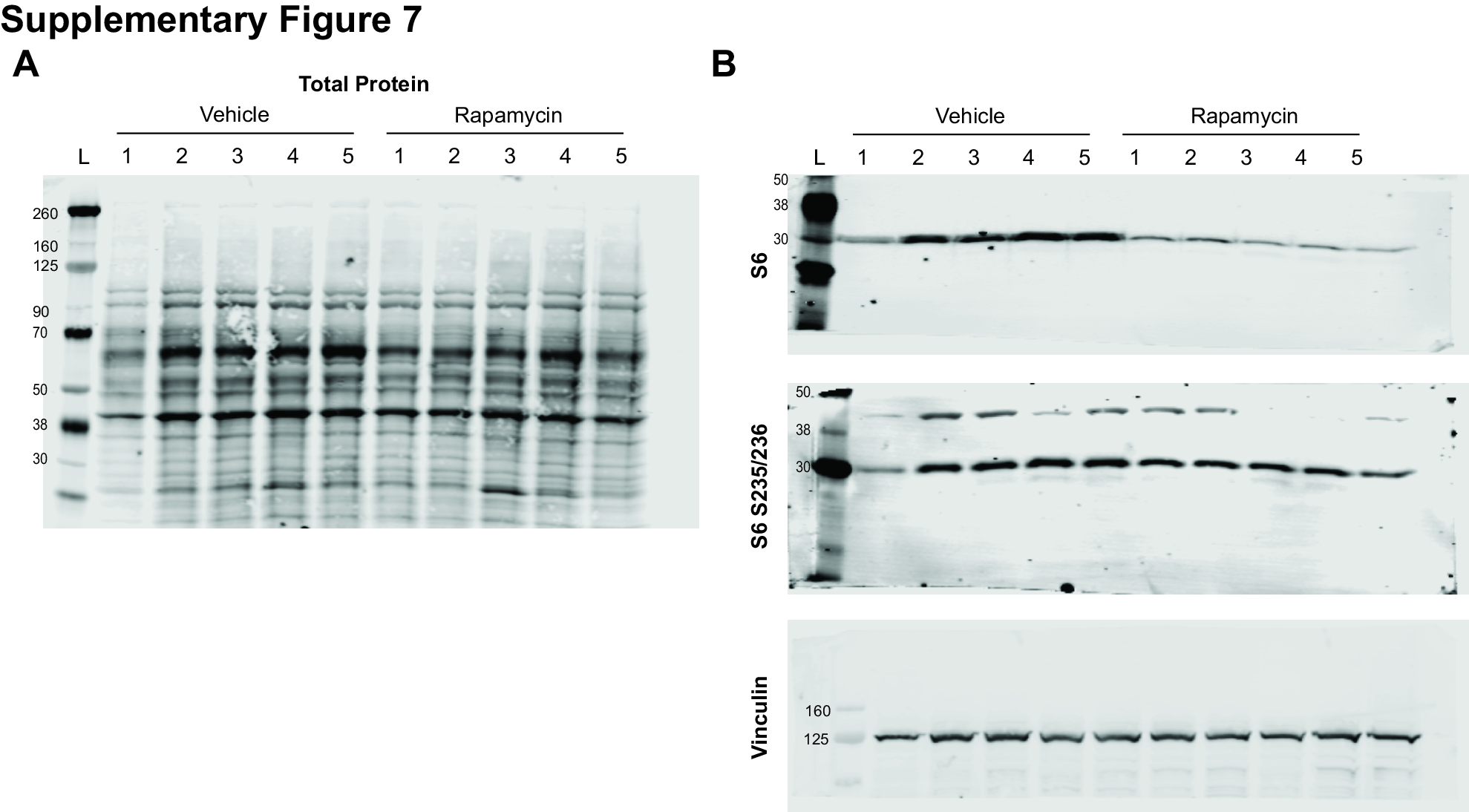

Supplement: Supplementary file 1 [file cancers-14-03374-s001.zip › Supplementary Figure S7.tiff]

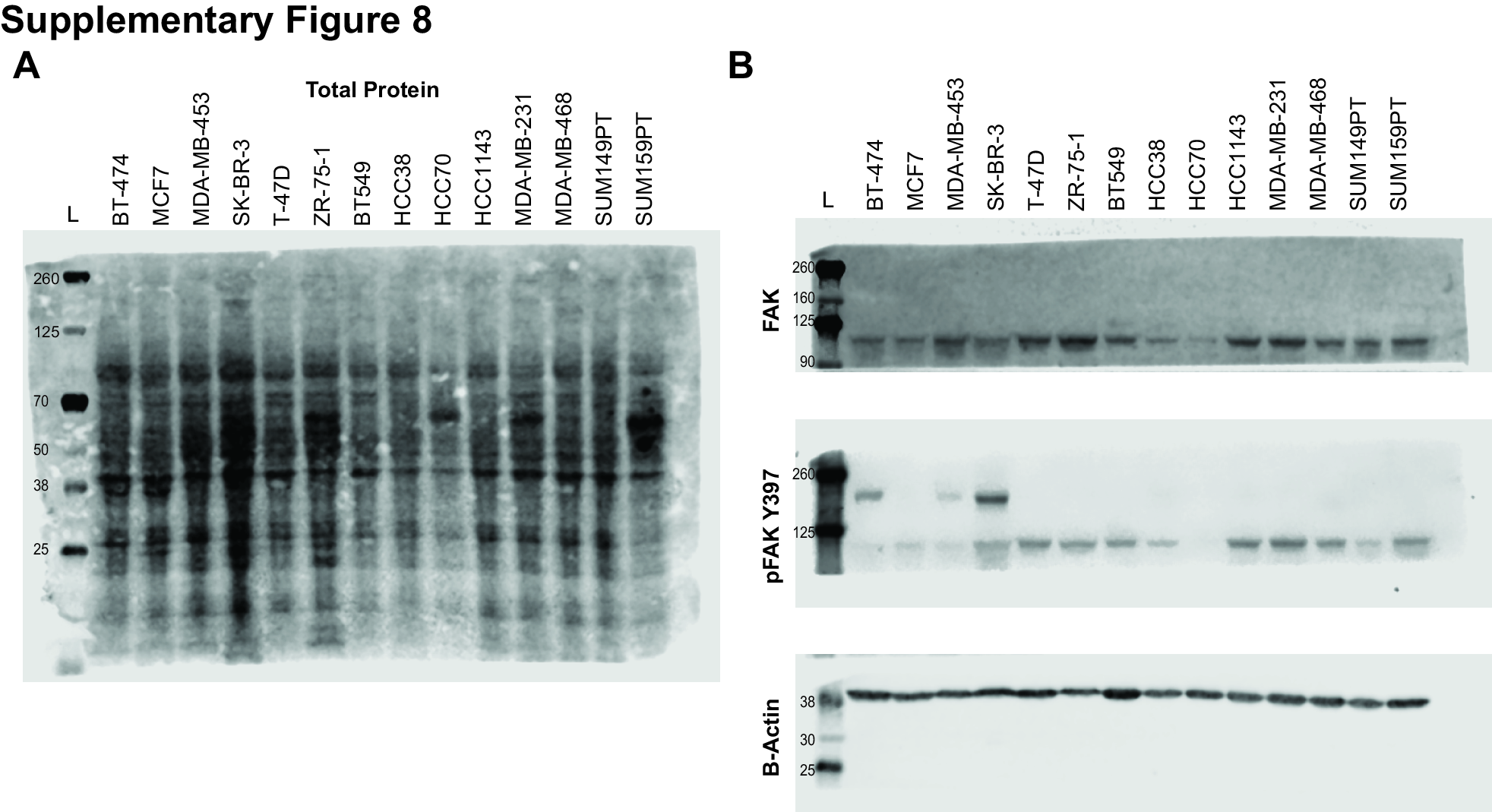

Supplement: Supplementary file 1 [file cancers-14-03374-s001.zip › Supplementary Figure S8.tiff]
